# Feeding brown fat: dietary phytochemicals targeting non-shivering thermogenesis to control body weight

**DOI:** 10.1017/S0029665120006928

**Published:** 2020-08

**Authors:** Carla Horvath, Christian Wolfrum

**Affiliations:** Institute of Food, Nutrition and Health, ETH Zurich, Schwerzenbach, Switzerland

**Keywords:** Brown adipose tissue, Browning, Energy expenditure, Phytochemicals, Weight management

## Abstract

Excessive adipose accumulation, which is the main driver for the development of secondary metabolic complications, has reached epidemic proportions and combined pharmaceutical, educational and nutritional approaches are required to reverse the current rise in global obesity prevalence rates. Brown adipose tissue (BAT) is a unique organ able to dissipate energy and thus a promising target to enhance BMR to counteract a positive energy balance. In addition, active BAT might support body weight maintenance after weight loss to prevent/reduce relapse. Natural products deliver valuable bioactive compounds that have historically helped to alleviate disease symptoms. Interest in recent years has focused on identifying nutritional constituents that are able to induce BAT activity and thereby enhance energy expenditure. This review provides a summary of selected dietary phytochemicals, including isoflavones, catechins, stilbenes, the flavonoids quercetin, luteolin and resveratrol as well as the alkaloids berberine and capsaicin. Most of the discussed phytochemicals act through distinct molecular pathways e.g. sympathetic nerve activation, AMP-kinase signalling, SIRT1 activity or stimulation of oestrogen receptors. Thus, it might be possible to utilise this multitude of pathways to co-activate BAT using a fine-tuned combination of foods or combined nutritional supplements.

Obesity is a major public health threat of the 21st century, both from an individual as well as from an economic point of view. Epidemiological data from extreme cases such as the USA with more than 70 % of adults being overweight or obese propose that obesity has sadly become the common or wild-type phenotype in certain nations^(1,2)^. Apart from the cosmetic imperfections arising from an obese phenotype, obesity-related metabolic complications and secondary diseases (different cancers, CVD, osteoarthritis, to name but a few) severely affect life quality and thus drive ongoing research efforts to identify weight lowering or stabilising pharmaceuticals. Possible approaches for the treatment of obesity were expanded by the discovery of physiologically relevant amounts of active brown adipose tissue (BAT) in adult human subjects^(3–5)^. Cold-induced BAT in human subjects negatively correlates with age and BMI^(3)^, while RMR shows a positive correlation^(4)^. The principal cell within BAT is the highly specialised brown adipocyte, which defends body temperature in a cold environment through non-shivering thermogenesis. This is enabled by uncoupling protein 1 (UCP1), localised to the inner mitochondrial membrane, which dissipates energy stored in the mitochondrial proton gradient generated by oxidative phosphorylation^(6)^. This catabolic process burns large amounts of fatty acids and glucose^(7–9)^ and therefore, active BAT likely serves as a nutrient sink to buffer excessive energetic intake or utilises energy reserves mobilised from white adipose tissue (WAT). In response to a cold stimulus, catecholamines are released from sympathetic nerve fibres that innervate BAT and function as inducers of the thermogenic programme. Other endogenous factors including fibroblast growth factor 21 (FGF21), natriuretic peptides, bone morphogenetic protein 8b, glucagon-like peptide 1, thyroid hormones or oestradiol are known modulators of BAT activity or recruitment^(10–12)^. In addition, the formation of brown-like adipocytes (beige/brite) within the WAT can be induced by cold acclimatisation or pharmacological means^(13,14)^. These beige/brite adipocytes express UCP1 and thereby extend the pool of thermogenically active cells. In summary, different routes are conceivable of how active brown adipocytes can assist in regulating metabolism and ultimately body weight (BW): (1) activation of existing brown cells within BAT, (2) formation of new brown cells within BAT or (3) induction of WAT browning.

Throughout history, human subjects relied on natural products to counteract disease, due to their richness in bioactive substances. Furthermore, many leading drugs are derived or isolated from natural sources and indispensable for human pharmacotherapy (e.g. morphine, metformin, sodium-glucose co-transporter 2 inhibitors, artemisinin, glucagon-like peptide-1 receptor agonist exendin-4). In view of the extent of the overweight/obesity problem, dietary constituents (phytochemicals) capable of tuning BAT activity could be used to reinforce weight loss programmes or to stabilise BW. A prime example of such a phytochemical is ephedrine, the active principle found in plants of the *Ephedra* genus, whose leaves are traditionally consumed as a tea. Ephedrine is a natural sympathomimetic and an herbal preparation from Ma Huang (72 mg ephedrine daily) significantly reduced BW (−4 *v.* –0⋅8 kg) and fat mass (−2⋅1 *v.* 0⋅2 %) in overweight to obese (BMI >29 and <35 kg/m^2^) patients (male *n* 6, female *n* 29) within 8 weeks when compared to placebo (male *n* 5, female *n* 28)^(15)^.

Obesity is caused by gradual fat accumulation due to a chronic positive energy balance. An American adult gains about 0⋅5–1 kg BW annually. The critical gap between energy intake and expenditure that advances weight gain is thus estimated to be as little as 92–682 kJ daily^(16)^. In turn, the total average human BAT mass is approximately 168 g and can burn 301 kJ within 2 h of cold-mediated activation^(17)^. This is exemplified by the synthetic β3-adrenergic receptor (AR) agonist mirabegron, which acutely activates BAT in fluorodeoxyglucose positron emission tomography–computed tomography scans and enhances RMR by approximately 837 kJ daily in men (200 mg) and by 10⋅7 % (628 kJ daily) in females^(18)^. Chronic mirabegron intake (100 mg daily for 4 weeks) elevates resting energy expenditure by 5⋅8 % (343 kJ daily) compared to baseline and increases BAT volume as well as BAT activity in women^(19)^. These treatment effects were notably more pronounced in women who possessed little BAT at baseline^(19)^. Thus, sustained β3-AR stimulation is potentially advantageous in subjects with elevated BW associated with lower BAT activity/volume.

It is pertinent to mention that sex-specific differences in human BAT activity, BAT mass and BAT detectability have been delineated in diverse studies, which likely modulate outcomes of pharmacological interventions. Female subjects are more frequently identified as BAT-positive in positron emission tomography–computed tomography scans (female:male ratio 2:1) and display higher BAT mass as well as BAT activity compared to men^(3,20–22)^. Various, yet not entirely resolved factors, such as the expression pattern of sex hormone receptors, levels of sex hormones, body size or the increased sensitivity of females to cold with distinct thermogenic responses might contribute to this sexual dimorphism^(23–25)^. Interestingly, BMI does not negatively correlate anymore with BAT activity or BAT mass in elderly men (43–82 years), while this correlation persists in female subjects (43–82 years)^(20)^. This suggests that older overweight/obese women might benefit more from BAT activation to regulate adiposity than men. Contrarily, Fletcher *et al*.^(26)^ could not confirm differences in cold-induced BAT activity or BAT distribution in healthy men *v.* women and the apparent higher BAT volume in men vanished after normalisation to body size^(26)^.

This review discusses a selection of phytochemicals present in food or drinks based on scientific evidence for their efficacy in human and animal models to affect weight or energy expenditure (EE). In particular, we provide an overview of how these substances modulate BAT activity or WAT browning at the molecular level.

## Stilbenes

Pterostilbene (PTS) is the dimethylated derivative of resveratrol (RSV) and its potential weight-lowering capacity has only recently gained attention. Interest in PTS rose when Rimando *et al*.^(27)^ demonstrated that PTS strongly induced PPARα activity in a rat hepatocyte cell line overexpressing a PPRE-luciferase reporter gene^(27)^. PTS at 100 μm displayed 2-fold higher PPARα induction than the pharmacologic PPARα agonist ciprofibrate. The anti-hyperlipidaemic activity of PTS was verified in hamsters where PTS-fortification of high fat diet (HFD; 25 mg PTS/kg food) led to lower total and LDL-cholesterol levels^(27)^. PPARα is a nuclear transcription factor, which is highly enriched in the liver as well as BAT *v.* WAT where it controls a myriad of genes involved in fatty acid uptake as well as β- and peroxisomal lipid oxidation^(28,29)^. The group of Maria Portillo examined the effect of PTS on adipose tissue in rat models of genetic and dietary-induced obesity^(30,31)^. In Zucker fatty rats, the daily oral application of 15 mg PTS/kg for 6 weeks resulted in significantly less total fat (10 % *v.* 13⋅2 %) compared to untreated animals^(30)^. During the dietary intervention, Wistar rats were fed an adipogenic high fat–high sucrose (20 % each) diet enriched with PTS to ensure an estimated daily intake of either 15 mg/kg or 30 mg/kg BW. Although no effect on weight gain or BW was observed, total adipose tissue mass was dose-dependently reduced in comparison with control animals (40⋅3 g, 36⋅6 g, 47⋅5 g)^(31)^. Moreover, enzymatic carnitine palmitoyltransferase 1α activity was higher in the liver while fatty acid synthase activity was blunted in WAT^(31)^. The described phenotype might result from an interplay between increased fatty acid oxidation due to higher fatty acid import into mitochondria via carnitine palmitoyltransferase 1α and reduced adipocyte lipogenesis due to low fatty acid synthase activity. Unfortunately, the only published human clinical trial with PTS as investigatory compound could not reproduce these findings from rats. Instead, 8 weeks of daily PTS ingestion (0, 100 or 250 mg daily) increased LDL-cholesterol and consequently total cholesterol levels in a cohort of male and female hypercholesterolemic Caucasian and African Americans^(32)^. Overall, a mild but significant effect of PTS on BMI was observed when participants were stratified for cholesterol medication. BAT possesses a tremendous oxidative capacity and relies heavily on substrate e.g. fatty acid oxidation to fulfil its thermogenic function. PPARα agonists are known to induce UCP1 expression in murine BAT^(33)^ and β-AR signalling increases PPARα expression in brown adipocytes^(34)^. To date, three publications have addressed the impact of PTS-fortified diet on WAT browning or BAT functionality in rodent models. Aguirre *et al*.^(35)^ found that 15 or 30 mg PTS/kg BW upregulated various brown and mitochondrial marker genes in male Zucker fa/fa rats when administered daily for 6 weeks^(35)^. These changes were accompanied by increased PPARα and UCP1 protein abundance in the BAT. Additionally, higher carnitine palmitoyltransferase 1α activity was measured in the intrascapular BAT lysate of PTS-rats compared to controls^(35)^. These molecular alterations imply that PTS enhances the oxidative capacity of BAT in genetically obese rats that translated to a beneficial phenotype as total adipose tissue weight and BW were reduced in PTS-rats^(35)^. This conclusion is substantiated by data from Nagao and co-workers^(36)^ who applied a 10× higher PTS dose (300 mg/kg BW daily) as a food supplement to male, genetically hyperphagic rats, which again caused lower body fat accumulation^(36)^. Compared to control-fed animals, PTS stimulated oxygen consumption (3⋅91 *v.* 3⋅69 litre/100 g BW daily) and enhanced EE (80⋅4 *v.* 77⋅1 litre/100 g BW daily) after 4 weeks of feeding^(36)^. The observed reduction in the respiratory quotient (0⋅82 *v.* 0⋅85) of PTS-treated rats was in agreement with higher lipid oxidation during the metabolically active dark phase^(36)^. Interestingly, Nagao *et al*.^(36)^ not only confirmed that PTS is a PPARα agonist, but also discovered that PTS at 50–250 μm increased sirtuin 1 (SIRT1) activity^(36)^. SIRT1 is a NAD+-dependent protein deacetylase and modulates the functional activity of various enzymes and transcription factors. Deacetylation of PPARγ by SIRT1 is crucial for browning of WAT as it enables the recruitment of the PPARγ coactivator PRDM16, which functions as a master regulator of the thermogenic gene programme in WAT^(37,38)^. In turn, food deprivation or pharmacological SIRT1 activation have been shown to blunt the PPARγ-dependent expression of lipogenic genes in WAT through SIRT1-mediated blockade of the PPARγ-coactivator NCoR^(39)^. This repressive effect on PPARγ promotes lipolysis and reduces fat storage in WAT^(39)^. SIRT1 additionally affects mitochondrial biogenesis by two converging mechanisms. First, SIRT1 deacetylates and activates liver kinase B1, an upstream kinase and activator of AMPK, which in turn phosphorylates PPARγ co-activator 1α (PGC1α)^(40)^. Secondly, SIRT1 directly deacetylates PGC1α^(41)^. Consistent with the relevance of SIRT1 in WAT browning, chronic PTS administration to HFD-fed mice (90 mg/kg daily) caused lower weight gain and a trend towards browning of the inguinal WAT as measured by upregulation of browning-specific marker genes (CIDEA, PGC1α, EBF2, PPARγ and TBX1)^(42)^. The transcriptional and morphological changes during WAT remodelling in response to cold exposure are independent of PPARα as PPARα knockout mice display equal browning when compared with wild-type animals^(43)^. Collectively, it seems that SIRT1 agonism by PTS is the dominant molecular trigger, which confers the physiological effects of long-term PTS consumption such as browning of WAT, enhanced oxidative capacity, lipid mobilisation and increased EE. The pharmacokinetic analysis of orally applied PTS (168 mg/kg BW) in rats revealed a high bioavailability of about 80 % with a peak plasma PTS concentration of approximately 30 μm^(44)^. This dosage corresponds to an approximated human equivalent dose of 27 mg/kg^(45)^ and up to 250 mg/kg BW of PTS is evidentially safe for human use^(46)^.

RSV attracted the interest of scientific community after Howitz *et al*.^(47)^ identified RSV as a powerful small molecule activator of SIRT1^(47)^. SIRT1 operates as one of the molecular regulators essential for the beneficial physiological effects of energetic restriction^(48)^. This discovery introduced the idea that RSV might be a natural substance able to ameliorate obesity by mimicking a low energy state. Lagouge *et al*.^(49)^ first demonstrated that a RSV-enriched (400 mg/kg BW daily) diet impairs weight gain in male HFD-fed mice^(49)^. RSV mice displayed enhanced oxygen consumption, improved cold tolerance during an acute cold challenge as well as increased mitochondrial content in BAT. A gene-enrichment analysis confirmed the induction of genes related to mitochondrial biogenesis and function after RSV therapy in muscle. The measured increase in EE was not attributable to spontaneous locomotor activity, suggesting that RSV modifies BAT functionality as a driver of adaptive thermogenesis. Another study explored the effects of lifelong RSV-containing HFD (0⋅04 % w/w) on metabolic health in middle-aged, male mice^(50)^. Here, RSV intake did not affect BW, possibly due to the lower concentrations used, but significantly attenuated the signs of ageing such as elevated fasting blood glucose levels, insulin resistance and pathological organ changes^(50)^. In a before–after study performed in six male, non-human primates, 4 weeks daily RSV ingestion (200 mg/kg BW) suppressed BW gain and enhanced RMR by 29 % compared to baseline^(51)^. In addition, the body temperature difference between the active phase and the hypothermic light phase was reduced, suggesting a thermogenic activity^(51)^. To date, direct, robust evidence for a contribution of BAT to the systemic effects of RSV is scarce. Two months of daily RSV feeding of male mice (400 mg/kg BW) on a standard diet led to lower epididymal and retroperitoneal adipose tissue weight, higher oxygen consumption and about 2-fold upregulation of UCP1 and SIRT1 mRNA expression in BAT of RSV mice^(52)^. The authors hypothesise that fat stores are mobilised to accommodate the increased fatty acid demand of more active BAT, which in turn lowers adiposity. However, no histological sections with UCP1-immunohistochemistry, UPC1 protein levels or mitochondrial parameters were presented to strengthen such a statement. The laboratory of Du^(53)^ demonstrated the formation of brown-like adipocytes in the presence of 10 μm RSV, when stromal vascular cells isolated from WAT were differentiated with a brown adipogenic cocktail^(53)^. These brown-like adipocytes upregulated an array of essential brown marker genes. This finding was affirmed in female CD-1 mice, where RSV (0⋅1 % in HFD) slowed weight gain and induced the same thermogenic gene set in the inguinal WAT when compared to controls^(54)^. Consistent with the known morphological features of browning, the inguinal WAT of RSV-fed mice exhibited adipocytes with multilocular lipid droplets and a shift towards smaller cells. RSV-treated mice and cells further displayed increased metabolic rates and enhanced lipid oxidation. More intriguingly, all the specified molecular alterations and the consequent physiological outcomes were not observed when experiments were repeated in the absence of AMPK^(54)^. Using the same experimental set-up, Wang *et al*.^(54)^ counted more brown adipocytes in histological BAT sections, which implies that RSV might provoke the formation of brown adipocytes, *in vivo*. In parallel, RSV feeding resulted in higher UCP1 and PRDM16 protein content as well as the augmented levels of phosphoAMPK^(54)^. Similar findings including reduced amounts of acetylated PGC1α were reported for the brown fat in male rats treated with 30 mg/kg BW^(55)^.

Contrary to the overwhelming amount of literature addressing the benefits of RSV in animal models, no human clinical trials exist that specifically use EE, BAT activation or weight management as readouts. A plethora of studies (supplementary material) conducted in different target groups with daily RSV doses from 75 up to 2000 mg did not detect any effects of RSV on key metabolic parameters, anthropometric measures or molecular downstream markers of SIRT1 activation^(56–58)^. In strong contrast stands a cross-over trial completed in obese, healthy men, where daily RSV (150 mg) intake for 30 d modestly mimicked the physiological effects resembling energetic restriction^(59)^. The participants had lower resting and sleeping metabolic rates (2–4 % lower EE), higher daily respiratory quotient as indicator for improved metabolic flexibility, higher SIRT1 and phosphoAMPK protein abundance in muscle biopsies plus elevated carbonyl cyanide-4-(trifluoromethoxy)phenylhydrazone-uncoupled respiration. This striking discrepancy in study outcome might be due to such factors as the extent of obesity, degree of insulin resistance, age, sex, sample size, parallel *v.* crossover design (seasonal impact) and blood RSV concentrations^(56–59)^.

The structural similarity between PTS and RSV implies that they might share the same molecular mode of action, SIRT1 agonism. Nonetheless, the methoxy groups in PTS protect it from hepatic metabolisation and possibly cause the high oral bioavailability of PTS (80 %)^(44)^. In contrast, the low oral bioavailability of RSV is one of its major drawbacks and might account for the poor reproducibility as well as comparability of human RSV trials. Although RSV is well absorbed in the intestine through passive diffusion (70 %), the phenolic groups are rapidly glucuronidated or sulphonated in the liver and leave only 1⋅5 % of RSV unmodified^(60)^. Notably, inter-species differences exist in the formation of glucuronidated RSV (g-RSV) metabolites. In rats and mice, 90 % of an applied RSV dose is glucuronidated compared to 65 % in human subjects and dogs. Lastly, enzymatic stereoselectivity leads to distinct ratios of g-RSV types: the dominant type (RSV-4’G) in human subjects and dogs is undetectable in mice and rats^(61)^. Consequently, findings from rodent studies are difficult to translate to human subjects and other models might be required to assess the physiological bioactivity/efficacy of RSV metabolites. The accepted difficulty in producing reproducible and meaningful effects with RSV in human subjects makes it currently unlikely to be upgraded from an over the counter nutritional supplement to a pharmacological remedy.

## Flavonoids

Quercetin is particularly abundant (16⋅5mg/g) in the onion peel extract (OPE; 16⋅5 mg/g), which is frequently used as a quercetin matrix in animal experiments^(62)^. OPE reduced diet-induced obesity by 6 % and visceral fat in male rats when added to HFD (3⋅6 g/kg food) for 8 weeks^(63)^. Ting *et al*.^(64)^ described an even more pronounced weight phenotype (20 % difference in weight gain) for a quercetin-rich supplement in male HFD-fed rats (185, 270 or 925 mg/kg BW daily), demonstrating that quercetin likely is the active ingredient in the OPE^(64)^. In an extensive study with Zucker fatty rats, quercetin was provided daily via the food (10 mg/kg BW) and profoundly reduced weight gain when combined with either standard diet (12 *v.* 26 %) or HFD (26 *v.* 33 %) over a 10 week period^(65)^. Quercetin-treated rats showed improved insulin sensitivity, reduced endothelial nitric oxide synthase protein and inflammatory TNFα release in the visceral WAT. Unfortunately, the aforementioned studies did not evaluate EE or physical activity as potential contributors.

In male mice, HFD enriched with 5 g OPE/kg for 8 weeks did not attenuate weight or fat mass compared to plain HFD but upregulated markers of WAT browning^(66)^. One possibility for the lack of a weight phenotype could be due to a stronger obese phenotype or different quercetin contents in the OPE (68 *v.* 276 mg/g dry weight). Browning of murine, male WAT following 12 weeks HFD feeding with pure quercetin (1 g/kg food) was confirmed by Kuipers *et al*.^(67)^. Classical BAT was not affected by the treatment and browning was insufficient to modulate BW or EE, in accordance with the results from Stewart *et al*.^(68)^ at a relatively high quercetin dose of 8 g/kg HFD^(68)^. A recent report^(69)^ describes browning of WAT alongside enhanced BAT-UCP1 content in male mice fed with HFD + 0⋅05 % quercetin^(69)^. Plasma norepinephrine concentrations were about three times higher in quercetin-exposed animals, which suggests an intensified sympathetic nervous activity as the underlying effector. Furthermore, protein kinase A (PKA) protein levels were elevated in subcutaneous WAT of quercetin mice. However, cAMP levels or phosphoPKA substrates, which are surrogate markers of adrenergic stimulation, were not determined. It remains to be shown whether these molecular adaptations translate into a distinct phenotype as body weight or EE were not measured^(69)^. Higher postprandial norepinephrine levels were also found in male calves fed quercetin (50 mg/kg BW) supplemented milk replacement for 1 week^(70)^. In human liver lysates, quercetin and its glycoside rutin showed catechol-*O*-methyltransferase (COMT) inhibitory activity at IC_50_ of 5⋅3 and 10⋅8 μm, respectively and could potentiate norepinephrine action^(71)^. Furthermore, quercetin dose dependently (1–250 μm) reinforced norepinephrine and isoproterenol-induced lipolysis in isolated male rat adipocytes by increasing cAMP concentrations^(72)^. Even though the effects of quercetin on BW are inconclusive, quercetin might assist in improving metabolic homoeostasis. Notably, inter-species differences in quercetin efficacy are obvious as most studies in rats showed a weight-lowering effect after 8–12 weeks of intervention, which was mostly absent in mice. Surprisingly, an extended quercetin feeding diet regimen over 12 weeks (1 g/kg HFD) in male mice added up to reduced terminal BW because of a lower adipose tissue mass^(73)^. In these mice, adipose tissue-resident macrophages and mast cells were significantly lowered, which caused attenuated visceral adipose tissue as well as systemic inflammation. Furthermore, UCP1 mRNA was upregulated in the BAT after quercetin treatment, suggesting a potential thermogenic effect^(73)^. At a molecular level, quercetin led to higher SIRT1 protein amounts and phosphoAMPK levels in the visceral WAT^(73)^. The anti-inflammatory property of activated SIRT1 is well established^(74,75)^. Induction of the SIRT1–liver kinase B1–AMPK pathway as a mediator of quercetin's anti-inflammatory activity was confirmed in bone-derived macrophages stimulated with lipopolysaccharides to mimic obesity-associated inflammation^(76)^. Under this condition, quercetin (20 μm) increased SIRT1, phosphoAMPK and phospho liver kinase B1 protein levels in parallel to a heightened intracellular AMP:ATP ratio^(76)^.

Although promising effects regarding BW and inflammatory states are evident in rodent studies, the current available human clinical trials do not reproduce this picture. An up to date meta-analysis covering doses from 100 up to 1000 mg daily and intervention durations from 2 to 12 weeks did not find any beneficial clinical effects of OPE or quercetin intake on human BW^(77)^. The absence of any human physiologically relevant effect is underlined by the studies summarised in the supplementary material, mainly performed in Korean subjects^(78–80)^.

It is striking that circulating quercetin concentrations reported in rodent studies appear much higher than in human subjects. In male mice, 5⋅3 g/kg BW daily quercetin related to 90 μm of fasting quercetin levels^(81)^ and a lower dose (100 mg/kg BW) to 10 μm after 12 weeks feeding^(73)^. In male and female human subjects, 150 mg daily over 5 weeks resulted in a mean fasting plasma quercetin concentration of 0⋅270 μm^(82)^, 500 mg daily over 12 weeks in about 1⋅3 μm^(83)^. Surprisingly, the mouse dose of 100 mg/kg BW translates to a human equivalent dose of 8 mg/kg BW and in human subjects, 500 mg daily reflects 6⋅5 mg/kg BW as calculated by the mean BW in this cohort. Consequently, although the doses lie in a comparable range, the circulating quercetin concentration in human subjects is roughly ten times lower. This comparison highlights potential inter-species differences in quercetin metabolism, absorption and excretion that affects final plasma concentrations and potentially the therapeutic outcome. Considering that human quercetin intake is safe up to 5 g daily^(84)^, clinical trials of extended duration (17 weeks) and at higher doses are required to conclude on the efficacy of quercetin in human subjects.

Luteolin has been extensively studied due to its anti-inflammatory activity and research focusing on weight loss following luteolin intake is in its early stages and limited to rodent studies. Distinct long-term dietary interventions with luteolin-enriched HFD (12 weeks, 0⋅01 % w/w or 16 weeks, 0⋅005 % w/w) significantly reduced BW gain in male mice^(85–87)^. This phenotype was due to reduced visceral and subcutaneous WAT accumulation with a lower proinflammatory state of the WAT (e.g. macrophage infiltration, lower M1:M2 ratio)^(85–87)^. Kwon *et al*.^(86)^ measured enhanced faecal lipid output in the luteolin group and higher rates of lipolysis in the WAT, which likely influenced adiposity^(86)^. Luteolin was shown to elevate PPARγ protein levels and transcriptional activity in 3T3-L1 adipocytes^(88)^. Modulation of PPARγ might contribute to the healthier adipose tissue expansion but cannot explain the reduction in adipose tissue mass as established pharmacological PPARγ agonists (thiazolidinediones) tend to increase fat mass^(89)^. It is reasonable to assume that the effect of luteolin on BW is polymodal and due to many physiological changes such as attenuated inflammation, reduced intestinal lipid absorption and more lipolytic WAT that fuels activated BAT. The latter assumption comes from a study by Zhang *et al*.^(90)^ who demonstrated higher EE in luteolin-HFD (0⋅01 % w/w) fed male mice compared to untreated mice^(90)^. The histological examination of the BAT revealed an increased density of UCP1+ cells in the luteolin group combined with an induction of the thermogenic programme at the mRNA and protein levels. Moreover, browning of subcutaneous WAT was detected. Interestingly, this phenotype was preserved when HFD was replaced by standard diet and proves the inherent thermogenic activity of luteolin^(90)^. At the molecular level, luteolin stimulated AMPK phosphorylation *in vivo* and the beneficial effects of luteolin on thermogenic genes was abolished in primary brown and white adipocytes when cells were pre-exposed to the AMPK inhibitor compound C. It appears that luteolin promotes an activated brown phenotype through the AMPK–SIRT1–PGC1α axis and human clinical studies are necessary for proof of concept/efficacy. Newest findings by Zhang *et al*.^(91)^ illustrate a significant role of mast cells in energy homoeostasis^(91)^. Genetic ablation or pharmacologic inactivation of mast cells in male mice amplified acute norepinephrine-triggered oxygen consumption and increased body temperature. In both conditions, massive browning occurred in the subcutaneous WAT together with higher UCP1 protein levels and the upregulation of thermogenic genes^(91)^. The authors claim that serotonin secretion from mast cells inhibits platelet derived growth factor receptor α + progenitor cell proliferation that can give rise to beige/brite adipocytes. In agreement with the anti-inflammatory features common to quercetin and luteolin as well as the attenuation of mast cells’ infiltration into WAT following the chronic consumption of these compounds, one can speculate that quercetin and/or luteolin enhance thermogenesis and systemic EE by WAT browning, due to lower mast cell-derived serotonin release.

## Catechins

Catechins are named after the catechu, an aqueous extract obtained from the wood of the acacia tree (*Senegalia catechu*) and rich in these polyphenolic substances. (−)-Epigallocatechin gallate (EGCG) and (−)-epigallocatechin are the most prevalent catechins in green tea infusions^(92–94)^. The first notion that green tea/catechin consumption might affect BW traces back to a study investigating the effect of a standardised green tea extract on 24-h EE in healthy men^(95)^. Surprisingly, three times daily intake of a combined caffeine (50 mg) plus EGCG (90 mg) preparation increased total 24-h EE by 3⋅5 % whereas the caffeine (50 mg) only group did not show any effects^(95)^. These findings were confirmed by Rudelle *et al*.^(96)^, who measured a comparable 4⋅6 % elevation (444 kJ daily) in the 24-h EE in lean men and women after a 3 d treatment diet regimen consisting of 282 mg EGCG and 300 mg caffeine daily^(96)^. Bérubé-Parent *et al*.^(97)^ next aimed to identify the necessary EGCG content in green tea capsules at a fixed caffeine dose (200 mg) to maximise the mixture's impact on 24-h EE in men^(97)^. Consistent with previous reports, three times daily ingestion of the mixture enhanced 24-h EE at any EGCG concentration (minimum 270 mg daily up to maximum 1200 mg daily); however, no significant dose–response was observed. It seems plausible that the thermogenic activity of the ingested green tea mimetic is mediated either solely by EGCG and/or by a synergistic action of caffeine and EGCG, whereas the effective amount of EGCG reaches a threshold beyond which no additional benefits on EE are to be expected. A 3 d trial focusing on the differential effects of EGCG or caffeine alone on EE in men (mean BMI 31) did not find any relevant impact at different EGCG concentrations (300 *v.* 600 mg) nor for caffeine (200 mg) or in combination^(98)^. Nevertheless, EGCG enhanced fat oxidation as determined by the respiratory quotient 2 h after an overnight fast by 7 % in the 300 mg EGCG group as well as in the postprandial phase, 2 h post-meal by 33 %. The contradictory results concerning EE may be due to the mode of administration e.g. capsules *v.* beverage, the ethnicity of the participants^(99)^, the degree of overweight/obese subjects and/or the utilised caffeine dose. Accordingly, EGCG-caffeine supplements seem to be more effective on weight loss or weight maintenance in subjects with low habitual caffeine intake^(99,100)^. A meta-analysis evaluating the influence of short-term EGCG consumption on EE and fat oxidation concluded that EGCG moderately boosts metabolic rate as indicated by significantly lower respiratory quotient and higher EE^(101)^.

A large body of literature^(102–107)^ (supplementary material) has examined whether the possible thermogenic activity of catechins translates to a weight loss phenotype during extended supplement interventions. Overall, the effect of catechins on weight maintenance or loss as measured by BW, BMI and waist circumference is significant when combined with caffeine whereas only a mild clinical impact can be implied based on the described effect sizes^(99,108)^.

The exact molecular events as well as the physiological targets, which trigger fat loss and elevated EE in response to catechin ingestion are still under discussion. However, the general consent supported by solid experimental evidence points at the activation and/or recruitment of BAT as likely effectors. In male rats, the supplementation of HFD with green tea extract (20 mg/g) over 2 weeks lowered body fat accumulation and boosted EE^(109)^. This phenotype was accompanied by higher BAT weight with increased total protein content, which is indicative for enhanced thermogenic BAT capacity. In turn, the positive impact of the green tea extract was abolished when the food was spiked with the β-AR inhibitor, propranolol^(109)^, suggesting that noradrenergic stimulation is required for the observed phenotype. In line with this, EGCG or tea catechins do not directly affect UCP1 mRNA expression levels in BAT. Imaizumi and coworkers^(110)^ reported that in an 8 week dietary intervention a minor (70 %) increase in UCP1 mRNA only when the tea catechins (40 % EGCG, 0⋅1 % caffeine) were administered in low fat diet to male rats^(110)^. Functionally relevant UCP1 protein abundance was not examined. In comparison, an EGCG (94 %) enriched green tea extract did not alter BAT-UCP1 mRNA levels in male mice after 4 weeks feeding with HFD although total body fat content was reduced^(111)^. An initial hypothesis proposed that the thermogenic action of EGCG originates from its ability to inhibit the COMT, resulting in higher norepinephrine levels in the postsynaptic cleft and therefore higher adrenergic input to BAT. COMT methylates, inactivates and initiates the degradation of catecholamines and thereby controls the availability as well as activity of these neurotransmitters. Catechins were shown to exert COMT-inhibitory activity in human subjects, mouse and rat liver extracts, although the determined IC_50_ values range from 70 nm up to 1, 15 or 55 μm depending on the substrate, catechin type or enzyme preparation used^(112–114)^. Nevertheless, the oral bioavailability of catechins is very low in men and women and plasma concentrations account for approximately 0⋅18 % of ingested catechins (400 mg) from black tea^(115)^. A pharmacokinetic analysis of daily green tea consumption (615 mg daily) detected peak plasma catechin levels 2 h post-prandially reaching roughly 85 ng/ml whereas EGCG levels were about 50 nm^(116)^. Additionally, catechins are estimated to be cleared from the body within 10–12 h after intake^(117)^. Considering these pharmacokinetic parameters, circulating catechin concentrations are unlikely to reach the required IC_50_ to exert a meaningful inactivation of COMT. Lorenz *et al*.^(118)^ proved that even a high dose of EGCG (750 mg) does not inhibit COMT activity *in vivo* when measured in human erythrocytes^(118)^.

An elegant, placebo-controlled crossover study performed in men^(102)^ revealed the direct involvement of BAT in the acute thermogenic effect of a catechin-rich beverage (615 mg). The participants were allocated to either low- or high-BAT groups based on their BAT activity measured with fluorodeoxyglucose positron emission tomography–computed tomography after 2 h of cold acclimatisation. Only the high BAT group displayed a significant increase in EE after the consumption of the catechin drink while there was no difference between the other groups^(102)^. Using the same technique, the authors found that prolonged daily catechin supply can increase cold-induced thermogenesis in the low-BAT group, which is paralleled by enhanced fat oxidation^(102)^. Also, in healthy Japanese women a daily 540 mg dose of catechins increased BAT density in the supraclavicular region^(103)^. These data indicate that catechins can recruit BAT to a physiologically relevant extent. Similar to capsinoids or capsaicinoids (described later), catechins might act through the transient receptor potential (TRP) vanilloid 1 (TRPV1) channels as first anticipated due to their astringent taste. TRPV1 are located on sensory neurons that signal to the central nervous system causing increased catecholamine release to stimulate BAT functionality. *In vitro*, EGCG has been shown to activate TRP channels in primary cultures of murine dorsal root ganglion neurons as well as in intestinal STC-1 cells^(119,120)^. The possible excitation of sensory neurons by catechins provides a plausible explanation for the observed threshold-dose of effective EGCG. Chronic exposure of TRP to high levels of capsinoids can induce receptor desensitisation and a comparable phenomenon might happen with high catechin use^(121)^. In addition, intestinal catechin levels are not affected by the low systemic bioavailability and are likely sufficient to elicit TRP. Interestingly, there seems to be a significant interaction between EGCG (100 μm) and a priming dose of the β3-receptor agonist ephedrine (0⋅1 μm) on oxygen consumption in male rats' BAT explants. The effect of EGCG on BAT respiratory rates was higher in the presence of ephedrine and was more profound with increasing ephedrine levels^(122)^. TRP channels are multimodal sensors that integrate distinct signals to fine tune their activity and modulate cellular events. PKA is the downstream effector kinase of β-AR signalling and known to positively affect TRPV1 efficiency^(123)^. Thus, EGCG signalling via TRPV1 might be effectively enforced by the β3-AR–PKA axis. However, up to date corresponding human data are lacking to support the hypothesis concerning catechins and TRP.

## Isoflavones: phytoestrogens

Soya represents the most dietary relevant isoflavone source with daidzein and genistein being the most prevalent forms and thus focus of this review. The positive nutritional profile of soya, specified by high protein, high isoflavone and low SFA contents, has long raised interest in weight management strategies, in particular due to favourable satiety and appetite control^(124)^. Genistein/daidzein share structural similarity to the female sex hormone 17β-oestradiol (E2), which not only masters female reproduction but also profoundly integrates into energy homoeostasis by controlling appetite as well as BAT and WAT functionality through central and peripheral action. Centrally, E2 acts on the ventromedial hypothalamus where it inhibits AMPK-signalling via estrogen receptor (ER) α and enhances sympathetic tone towards BAT and upregulates BAT UCP1 and PGC1α^(125)^. Similar to postmenopausal women, loss of E2 signalling in ovariectomised rats stimulates hyperphagia with resultant weight gain. E2 administration reverses this phenotype by increasing EE through the ERα–sympathetic nervous system–BAT axis and decreasing food intake. E2 is a membrane permeable, hydrophobic molecule and able to tune brown adipocyte function directly by signalling through ERα, ERβ or g-protein coupled receptor 30^(126,127)^. In cultured brown adipocytes isolated from male mice, E2 increases β3-AR mRNA expression and exerts the opposite effect on inhibitory α2-levels, which could potentiate sympathetic inputs^(128)^. E2 further promotes mitochondrial biogenesis by inhibiting phosphatase and tensin homolog, causing more active AKT-signalling and nuclear translocation of nuclear respiratory factor 1, the head of mitochondrial gene transcription^(129,130)^. Therefore, the question arises whether the oestrogenic potency of dietary isoflavones is effective at shaping energy homoeostasis to a physiologically relevant extent such as weight loss or stabilisation.

In male mice, HFD supplementation with the isoflavone-rich fraction (9 g/kg) of the Kudzu flower lowered weight gain as well as WAT and BAT weight after 7 weeks. Histological analysis of BAT from treated mice revealed more UPC1+ cells, suggesting that isoflavones activate BAT to burn more fatty acids^(131)^. Comparable findings were reported for male CD-1 mice fed with a soya-enriched (25 % w/w), high phytoestrogenic diet (HP; 150 ppm daidzein, 190 ppm genistein) when compared to a soya-free, low phytoestrogenic diet^(132)^. This dietary intervention resulted in a 7⋅6 % lower weight gain in HP mice compared to low phytoestrogenic controls with significantly reduced adipose tissue mass. The BAT of HP mice was denser, concomitant with higher EE and apparent cold resistance during an acute cold challenge^(132)^. As anticipated from the efficacy of exogenous oestradiol administration to ovariectomised rats, an isoflavone-rich diet (200 μg/g) prevented the severity of weight gain in these animals post-surgery^(133)^. Similar findings, including the upregulation of UCP1 protein levels and higher plasma T3 concentrations, were reported by Lephart *et al*.^(134)^ using a diet with an isoflavone content of 600 ppm in male rats^(134)^. They showed that dietary isoflavone ingestion is effective in a model of low E2 and enhances metabolic rates at least partially due to brown fat activation. Several groups investigated the potency of the individual isoflavone types on weight development and EE in various models. In lean or obese rats, enrichment of high fat or standard diet with daidzein (50 mg/kg BW, 14 d) stabilised BW or induced weight loss compared to controls^(135)^. Dose-dependent weight loss after daidzein intake (30 d) was also observed in obese male mice at 50 mg/kg BW (42⋅9 to 33⋅7 g) and at 100 mg/kg BW (42⋅5 to 32⋅4 g) but not at 25 mg/kg BW when compared to vehicle controls^(136)^. Although EE was not measured, UCP1 immunofluorescence imaging showed higher UCP1 content in the BAT of the daidzein-exposed group^(135)^. *In vitro*, daidzein stimulated hormone sensitive lipase mediated glycerol release from primary adipocytes from male rats indicating that elevated adipocyte lipolysis supports daidzein-induced weight loss^(136,137)^. Elevated fatty acid generation in brown adipocyte could enhance EE by directly activating UCP1. Additionally, daidzein was shown to inhibit phosphodiesterase activity at an IC_50_ of 50 μm^(138)^, a critical enzyme which arrests β-AR signalling by inactivating cAMP.

The literature is more extensive for the efficacy of genistein, probably due to the higher oestrogenicity^(139,140)^. In female mice, 8 weeks genistein treatment with HFD (0⋅25 % w/w) resulted in reduced weight gain and lower WAT expansion than in control HFD animals. Within the WAT, brite adipocyte marker genes were upregulated, which was probably centrally controlled^(141)^. Urocortin-3 (UCN3) was among the differentially regulated hypothalamic genes when HFD *v.* HFD + genistein and HFD *v.* control animals were compared. UCN3 is an anorexigenic peptide and a known player in energy homoeostasis. Male UCN3 transgenic mice are protected from HFD-induced weight gain^(142)^ and intracerebroventricular UCN3 administration to male rats provoked thermogenesis^(143)^ as well as higher BAT UCP1 expression levels^(144)^. In another recent study, genistein-exposed (0⋅2 % w/w) male mice displayed higher EE and the appearance of UCP1+ cells within the subcutaneous WAT compared to chow-fed controls, confirming the role of genistein in adipose browning^(145)^. We confirmed a cell autonomous effect of both genistein and daidzein in mature immortalised brown adipocytes, which enhanced UPC1 promoter activity in a UPC1-driven luciferase assay and led to higher UCP1 activity as measured by UCP1-immunofluorescence intensity^(146)^. In 4-week old male and female mice, daily administration of genistein (200 mg/kg BW) for 15 d lowered body weight by 4 and 4⋅3 %, respectively due to reduced food intake as well as reduced adipogenic gene expression mediated by ERβ^(147)^. In human fetal brown fat, ERα is the dominant oestrogen receptor type^(148)^ but the ratio is unknown in adulthood. Ligand assays unveiled higher affinity of genistein and daidzein for ERβ than ERα, demonstrating that isoflavones preferentially activate the oestrogen-response element via ERβ. The EC_50_s of ERα for genistein (15 μm) and daidzein (>300 μm) are supraphysiological and beyond circulating levels achievable^(149)^ with dietary isoflavone supplementation (100 mg isoflavone capsules daily; thereof 14 mg daidzein and 3–4 mg genistein)^(150)^. Given these concentrations, it is unlikely that isoflavones exert their physiological impact through ERα, fitting to the overall impression that E2-treatment via sympathetic nervous system-ERα signalling elicits more pronounced alterations in energy homoeostasis. Nevertheless, an ERβ-agonist (β-LGND2) conferred anti-obesity effects in male HFD-fed mice^(151)^. Pharmacological ERβ activation (30 mg/kg BW daily, s.c.) elevated EE and acute cold tolerance by 14 %. The thermogenic β-LGND2-mediated response culminated in a fat mass loss of 50–60 % compared to control animals. These beneficial effects were dramatically blunted, although not absent, in ERβ-knockout mice. At the molecular level, β-LGND2 stimulated WAT browning and enhanced the mitochondrial respiratory capacity of white adipocytes.

No human clinical trial so far included EE or BAT activation as outcome measures. Most reports^(152–158)^ (supplementary material) focus on anthropometric measures and body composition as surrogates for alterations in energy homoeostasis. The interpretation of human trials investigating the efficacy of isoflavone mixtures or individual compounds is complex as interactive factors such as sex, pre-/postmenopausal state, habitual isoflavone exposure/ethnicity and interindividual variation in metabolising enzymes (hepatic or intestinal, gut microflora) need to be taken into account. Within their food matrix, isoflavones exist usually as glycosides (genistin and daidzin)^(159)^. However, they are rapidly hydrolysed to their aglycones after ingestion and undergo intestinal and hepatic metabolisation generating distinct metabolites, among others equol^(159)^. Equol is built from daidzein by intestinal bacteria and is more bioactive than its precursor^(160)^. Surprisingly, all experimental animals are able to generate equol but not every human subject is an equol producer^(161)^. Subject to differences in the gut microbiome, only 20–35 % of Western individuals are equol-producers, whereas in the Asian region the rate is 50–60 %^(162–164)^. There is also a sex and age bias towards postmenopausal women in the published trials. In this target group, dietary isoflavones might simply aid in minimising or compensating the effects of declining endogenous oestrogen levels and mitigate postmenopausal weight gain. Presently, two meta-analyses dissect in depth the efficacy of isoflavones on body composition and weight development. These studies provide a clearer view on the effects in different subpopulations or influencing factors such as dosing, weight status or trial duration. The meta-analysis from Zhang *et al*.^(165)^ concentrates specifically on postmenopausal non-Asian women and included nine trials for BW^(165)^. Soya isoflavone supplementation was associated with a significant lower BW and subgroup analyses revealed that the difference is more pronounced in normal weight than obese women as well as with lower doses (<100 mg daily)^(165)^. A second analysis performed by Akhlaghi *et al*.^(166)^ includes both sexes, all ethnicities independent of age and comprised forty-seven trials with soya and seventeen trials with isoflavones as investigational subjects^(166)^. BMI in response to isoflavone intake tended to be lower (*P* = 0⋅085), especially with lower doses and shorter trial length. The subgroup analysis suggests that postmenopausal and Caucasian women likely benefit more from isoflavone intake. Contrarily, soya intake does not affect BW, fat mass or waist circumference. The latter two are also not modified by isoflavone supplementation^(166)^. Taken together, it seems that isoflavones have a mild beneficial effect on BW development and seem to be particularly beneficial in postmenopausal women to account for the reduced endogenous oestrogen levels. Although lower doses below 100 mg daily are more effective, they still exceed the amounts achievable through normal dietary sources, even in populations with high daily isoflavone intake (25–50 mg)^(167)^. Thus, isoflavone fortification or supplements would be required to fulfil a physiological relevant intake.

## Alkaloids

Alkaloids exert a plethora of pharmacological activities crucial for human pharmacotherapy (e.g. morphine, quinine and vinblastine)^(168)^ and the alkaloids capsaicin (CAP) and berberine (BBR) have been shown to affect BAT activity or induce browning of WAT.

Capsaicinoids, especially CAP, confer the pungent sensation of red chilli from the *Capsicum* genus^(169,170)^. The thermogenic properties of CAP-rich food are reflected in a variety of physiological responses such as elevated body temperature, flushing, vasodilation and the concomitant onset of cooling mechanisms such as sweating. CAP stimulates afferent sensory neurons involved in thermo- and nociception by binding to TRPV1, which increases intracellular Ca^2+^ concentrations that trigger membrane depolarisation^(171)^. Many groups have examined the effect of acute or chronic capsaicinoids/capsinoids on EE, fat oxidation, weight management and the contribution of thermogenically active BAT, with diverging outcomes. Early studies investigating the influence of spicy foods using chilli sauce (3 g) or red pepper (30 mg CAP) on metabolic rates in men reported significant increases in postprandial EE by 25–32 % compared to control meals^(172,173)^. This metabolic effect was abolished when the participants were pre-treated with the β3-AR antagonist propranolol^(173)^, indicating the involvement of sympathetic nerve activity. Higher sympathetic:parasympathetic nervous system activity was confirmed in Caucasian men after the ingestion of a red pepper-spiked (6 g) appetiser compared to a control snack^(174)^. In contrast, Smeets *et al*.^(175)^ did not find elevated diet-induced thermogenesis after a CAP-containing lunch (approximately 5⋅15 mg) nor altered substrate oxidation in men and women^(175)^. However, the effect size might be too small, given a 6-fold lower CAP dose. Another placebo-controlled trial in men failed to identify a significant difference in the RMR of healthy subjects treated acutely with increasing amounts of dihydrocapsiate (0, 3 or 9 mg)^(176)^. *In vitro*, CAP agonises TRPV1 at an effective dose (EC_50_) of 50–100 nm. Contrarily, the EC_50_ of capsiate and dihydrocapsiate are 580 and 670 nm, respectively^(177,178)^. This discrepancy in potency between CAP and capsinoids combined with varying doses might resolve the absence of an effect in the dihydrocapsiate trial. A meta-analysis^(179)^ covering the efficacy of capsiate and CAP intake on EE in human subjects reveals that capsiate enhances lipid utilisation and EE while CAP is only effective on fat oxidation. The effects of CAP on EE was only relevant in high dose trials (135–150 mg CAP), when CAP intake was stratified for dosage^(179)^. For both substances, sympathetic nervous activity was stimulated where this outcome was recorded^(179)^. Orally administrated CAP and capsiate (10 mg/kg BW) equally boosted oxygen consumption and serum catecholamine levels in mice (unknown sex) when compared to untreated animals^(180)^. Kawabata *et al*.^(181)^ identified that the acute intragastric administration of capsinoids or CAP (10 mg/kg BW, each) to male mice increased metabolic rate and stimulated fat oxidation in wild type, but not TRPV1 knockout mice, demonstrating the necessity of the vanilloid receptor as thermogenic mediator^(181)^. Adrenergic input is a potent activator of the thermogenic BAT programme as well as subcutaneous WAT browning and TRPV1 is expressed in both adipose types. Additionally, murine TRPV1 levels are upregulated in both BAT and WAT following a CAP-enriched diet^(182–184)^. Therefore, it was soon hypothesised that enforced BAT functionality or a bigger brown/brite adipocyte pool contributes to the physiologic effects of CAP. A direct effect of CAP on BAT activity was already implied in 1988, when Yoshida *et al*.^(185)^ demonstrated that the intramuscular injection of 3 mg/kg CAP to rats elevated intrascapular BAT temperature locally and increased mitochondrial oxygen consumption^(185)^. Similarly, 3 h intragastric CAP or capsinoid administration by Kawabata *et al*.^(181)^ (see earlier) heated up core and local BAT temperatures only in wild-type but not TRPV1 null mice^(181)^. A role of BAT in capsiate action is further supported by an acute capsiate exposure for 30 min that upregulated UCP1 mRNA levels in the BAT and evolved to higher UCP1 protein abundance in the BAT from male mice after 2 weeks of capsiate supplementation^(186)^. A detailed study by Baskaran *et al*.^(182)^ substantiates the interplay between CAP, active BAT and TRPV1 in male mice^(182)^. The addition of CAP to HFD (0⋅01 % w/w) over a 32 week intervention period prevented weight gain by almost 35 % compared to HFD controls and led to a final BW comparable to mice on standard diet. This impressive BW phenotype was not observed in TRPV1-knockout animals with the same diet regimen. Other rodent studies with males addressing obesity prevention by the supplementation of HFD (0⋅014 %, rat)^(187)^ or standard diet (10 mg/kg, mouse)^(180)^ with CAP or capsiate up to 14 d equally reported lowered weight gain and stimulated fat loss from WAT compared to controls^(180,187)^. Regarding obesity treatment, CAP feeding attenuated additional weight gain in already obese male mice during a 10 week follow-up period (36⋅5 *v.* 41 g), resulting in reduced adiposity^(184)^. Furthermore, CAP feeding lowered circulating TAG levels, which put forward the idea that CAP mobilises lipids from adipose depots and enhances lipid oxidation, likely in the BAT^(180,187)^. Apart from elevated EE^(180,182,183)^, the RER of CAP-HFD male mice was significantly higher^(182,183)^ and points towards increased carbohydrate oxidation. Others found enhanced fat^(181,186)^ and reduced carbohydrate^(181)^ oxidation after CAP-administration, which underlines a higher metabolic flexibility in these animals. Indications for metabolically more activated CAP-BAT were further evident in the heightened glycerol release from this tissue as surrogate for lipolysis in comparison with HFD-BAT^(182)^. The lipolytic principle of CAP is very likely mediated by augmented catecholamine secretion and signalling through the β-AR–PKA–hormone-sensitive lipase pathway. Acute stimulation of brown adipocytes from male CAP-HFD mice with 1 μm CAP dramatically increased intracellular calcium influx, which was blunted in TRPV1-lacking adipocytes^(182)^. A downstream analysis of the molecular events revealed that CAP activates TRPV1 leading to higher calcium levels and the activation of CaMKII, which in turn phosphorylates and activates AMPK. AMPK stimulates SIRT1 activity triggering the deacetylation of PGC1α and PPARγ as ultimate enhancers of BAT functionality. Baskaran and coworkers^(183)^ demonstrated the browning of the inguinal WAT in response to CAP diet, based on the same molecular mechanism^(183)^.

Human clinical trials evaluating the long-term effect of CAP/capsiate on weight development are rare (supplementary material). A study by Janssens *et al*.^(188)^ implies a role of CAP supplementation (2⋅56 mg/meal) in weight maintenance after weight loss in male and female subjects. Accordingly, the acute thermogenic effect of CAP could be sufficient to compensate for the reduction in EE that goes along with weight loss and energetic restriction^(188)^. In a US placebo controlled-trial with obese male and female participants (mean BMI 30 kg/m^2^) the daily ingestion of 6 mg capsinoids over a 12 weeks trended in a BW change (0⋅9 *v.* 0⋅5 kg) and significantly reduced visceral adiposity^(189)^. However, more of these long-term studies with higher doses or in relation to weight stabilisation after weight loss are required to delineate the pharmacological potential of capsinoids. A recent crossover study directly captured BAT-activity during an acute capsinoid (12 mg) trigger in men and women by luorodeoxyglucose positron emission tomography–magnetic resonance in comparison with cold-exposure using a cold vest (14⋅5°C). The mean standardised uptake value (SUV) for the capsinoid group did not reach the accepted threshold (SUV = 2) to classify a subject as BAT-positive, contrary to the cold-exposed group (mean SUV = 2⋅9). Only after an adjustment of the grey scale values, a mild uptake of glucose was notable in the capsinoid group. Nevertheless, the mean EE post-capsinoids administration was higher compared to baseline levels and this increase was significantly greater in subjects allocated as BAT-positive based on cold-exposed BAT detectability^(190)^. Unfortunately, this study lacked a placebo control although the capsinoids were ingested as capsule containing additionally a mixture of rapeseed oil and medium chain TAG. A placebo-controlled, crossover trial with eighteen Japanese men verified an acute increase in EE after capsinoids intake (9 mg) in BAT-positive subjects when compared to placebo controls and BAT-negative individuals^(191)^.

In conclusion, it appears that the physiological outcomes associated with spicy food/CAP ingestion are the sum of four primary events: (1) enhanced sympathetic activity due to the stimulation of vagal afferent neurons and catecholamine levels, which (2) promote lipolysis in WAT and BAT and (3) activate non-thermogenesis in BAT; (4) the direct, cell autonomous trigger on adipocytes via TRPV1–AMPK–SIRT1 to enhance the thermogenic phenotype. The documented bioavailability (50–90 % absorption) of capsaicinoids is high^(181)^. In mice, circulating plasma CAP concentration 1 h after an oral dose of 10 mg/kg BW reaches approximately 3⋅8 μm^(192)^. This value falls into a concentration range that induced browning *in vitro* in 3T3-L1 and indicates that circulating CAP levels are sufficient to elicit direct cellular effects^(193)^.

BBR is arguably the most prominent known phytochemical capable of modulating BAT function and several preclinical studies have delivered interesting outcomes regarding the therapeutic impact of BBR on obesity-associated metabolic diseases. BBR has a long history of use in traditional medicines and interest in the metabolic side of BBR awoke only in 2004 when the blood cholesterol-lowering activity of BBR in hypercholesterolemic patients was published^(194)^. Another study underpinned the therapeutic potential of BBR in genetically and fat-induced obese rodents^(195)^. In male db/db mice a daily dose of BBR (5 mg/kg BW, i.p.) caused a 13 % weight loss with a 10 % reduction in fat mass within 26 d of treatment alongside with improved whole body metabolic homoeostasis^(195)^. The same physiological effects were observed in male HFD-fed rats treated with either vehicle or BBR (380 mg/kg BW, orally)^(195)^. In WAT, BBR downregulated lipogenic genes while oxidative and genes regulating mitochondria formation/function were enhanced in muscle and BAT (PPARα and PGC1α)^(195)^. In 3T3-L1 cells, they subsequently identify that BBR acts as an AMPK activator, stimulating the phosphorylation of AMPK and acetyl-CoA carboxylase^(195)^, possibly by inhibiting complex I causing a rise in the cellular AMP:ATP ratio^(196)^. These findings motivated further research exploiting the effects of BBR on BAT activity as AMPK plays an accepted role in BAT-mediated thermogenesis and EE^(197–199)^. Zhang *et al*.^(200)^ first reported a thermogenic effect by BBR^(200)^. Apart from the established metabolic and weight improvements, BBR-therapy to male db/db mice (5 mg/kg BW, i.p.) increased rectal temperature (about 1⋅5°C), consistently enhanced metabolic rate and defended body heat loss during cold exposure compared to vehicle treatment. Most strikingly, BBR dramatically enforced BAT thermogenesis directly as reflected by higher 18-FDG uptake monitored using micro-positron emission tomography–magnetic resonance. This activated BAT state was supported by reduced BAT mass, higher mitochondrial content and molecular evidence such as enhancement of the BAT-specific mRNA and protein signatures (PGC1α, UCP1, nuclear respiratory factor 1 and carnitine palmitoyltransferase) through activation of AMPK^(200)^. BBR likewise stimulated browning of the inguinal WAT. Interestingly, immunohistological analysis revealed higher tyrosine hydroxylase levels in the WAT and BAT, suggesting the contribution of augmented sympathetic output to the observed phenotype. When the animals were housed under thermoneutral conditions (30°C) to minimise BAT function, the beneficial effects of BBR administration were blunted, which strongly supports the necessity of active sympathetic activity as physiologic mediator of BBR activity^(200)^. WAT-browning and elevated EE were also noted in male mice on HFD treated for 5 weeks with BBR (5 mg/kg BW i.p.) or vehicle^(201)^. Additionally, BBR upregulated hepatic FGF21 gene expression resulting in a concomitant elevation in plasma FGF21 levels, which was dependent on activated SIRT1, a known regulator of FGF21 transcription^(202)^. FGF21 was shown to promote WAT browning as well as the thermogenic BAT programme elsewhere and might depict an alternative mechanism of action of BBR^(203,204)^. Wu *et al*.^(205)^ clearly show the requirement of intact AMPK signalling in adipose tissues for an effective BBR treatment as the physiological responses to BBR administration were absent in adiponectin-Cre driven AMPK-floxed male mice^(205)^.

In the same work^(205)^, the effect of 1 month dietary BBR supplementation (3× 500 mg daily) on human BAT activity was examined in overweight individuals. Using cold-stimulated fluorodeoxyglucose positron emission tomography–computed tomography imaging, they prove that human BAT is activated by BBR as mean SUV (2⋅6–3⋅3), BAT mass (14⋅1–25⋅5 cm^3^) and activity (103⋅1–228⋅2 ml × SUV_ave_ × g/ml) were all significantly increased in a paired before–after analysis. Patients (*n* 2) with no detectable BAT before treatment were not BAT-positive after 1 month of BBR intake. Thus, BBR is not sufficient to stimulate BAT formation or induce dormant brown adipocytes. The percentage change of BAT activity before–after BBR negatively correlated with the measured changes in BW and highlight the potency of BBR to combat overweight by means of BAT activation. A future pharmacological application of BBR to tackle obesity and associated metabolic complications is supported by additional human clinical trials. An open-label study in China investigated the effect of a lifestyle intervention (LSI) with or without daily BBR (3× 500 mg) or pioglitazone (15 mg) treatment on BW and metabolic health in individuals suffering from non-alcoholic fatty liver disease with diabetes or impaired glucose tolerance^(206)^. At the end of the intervention, the BBR group lost significantly more weight (−4⋅29 *v.* –1⋅44 kg) and waist circumference (−4⋅84 *v.* –2⋅14 cm) than LSI only, but also when compared to LSI plus pioglitazone. As there was no significant difference between LSI + pioglitazone and LSI + BBR on insulin sensitivity, glucose tolerance or glycated Hb levels, BBR improved glucose homoeostasis comparably to a known antidiabetic remedy with the additive effect of weight loss^(206)^. BBR was equally effective in placebo-controlled trials with Chinese type 2 diabetic and dyslipidaemic patients (1 g daily)^(207)^ or poor glycaemic control (1⋅5 g daily)^(208)^. The BBR group displayed significant improvements in BW (66⋅4 *v.* 70⋅5 kg), BMI (24⋅3 *v.* 25⋅4 kg/m^2^) and glycaemic control (fasting blood glucose 5⋅6 *v.* 6⋅4 mm/, HbA1c 6⋅6 *v.* 7⋅3 %) compared to controls at the end^(207)^. In a small pilot study (*n* 7) with obese Caucasian individuals over 13 weeks (1500 mg BBR daily) moderate changes in BW (2⋅3 %), BMI (2⋅9) and fat content (3⋅6 %) were determined compared to baselines values. Although these changes seem promising, they did not reach statistical significance likely due to the small sample size^(209)^. The available studies centring on BW control or weight loss are sparse and dominated by Asian subjects (supplementary material). Nevertheless, the reported outcomes are overly encouraging and underline the need for further efforts aiming at different ethnicities and larger sample sizes. Furthermore, it would be interesting to study whether BBR enhances EE in human subjects. One of the current drawbacks is the extremely poor bioavailability of BBR in human and animal studies. In human subjects, oral BBR intake (300 mg daily) resulted in a mean plasma BBR concentration of 0⋅3 ng/ml and an acute BBR (400 mg) challenge led to a maximal concentration of 0⋅4 ng/ml^(210)^. BBR combined with polyethylene glycol enhanced intestinal BBR absorption in rats by a factor of 3, indicating that the pharmacokinetic properties of BBR are adjustable with appropriate encapsulation strategies^(211)^. Improved BBR bioavailability is desirable as it affects the effective treatment dose and might reduce some of the mild side effects (e.g. constipation and intestinal discomfort) observed with the currently employed BBR dose of 1500 mg daily. Alternatively, the efficacy of the more absorbable analogue dihydroberberine with five times higher bioavailability than BBR in rats could be investigated for human use^(212)^.

## Conclusions

BAT is a remarkable organ with the capacity to ignite its thermogenic programme to ‘waste’ energies in response to environmental or endogenous cues. From the afore-mentioned reviewed phytochemicals we can draw several conclusions. First, preclinical evidence of efficacy from animal studies is limited in human trials and the reported effect sizes in human subjects tend to be small. Differences in the gut microbiome and metabolising enzymes influence whether, how and to what extent a phytochemical is altered from its prodrug state to either a functionally more or less active metabolite that reaches the systemic circulation and target sites. Animal experiments are performed in inbred strains with near identical genetic background whereas in human subjects the interindividual variability is more pronounced. This is intensified by differential study designs (duration, dose, ethnicity, formulation, etc.) that might cause the large variability between human studies. Secondly, sex as biological variable adds extra complexity to the interpretation of results, which demands critical attention. Out of the thirty-nine human randomised controlled trials listed in the supplementary material, fifteen included male and female subjects, thirteen only males and nine only females. Females were notably overrepresented in trials with isoflavones due to their oestrogenic potential. No studies with ‘females only’ are listed for PTS, BBR and capsaicinoids and only one for catechins. The vast majority of the cited rodent studies were performed in male animals due to prevailing belief that females' hormonal cycle increase heterogeneity and results derived from male animals are translatable to females^(213)^. In contrast, the female gonadal WAT is more innervated by sympathetic nerves and responds more effectively (browning) to a β3-AR agonist compared to male gonadal WAT^(24)^. Similarly, the BAT of female rats displays a more multilocular morphology, a denser mitochondrial network with higher UCP1 protein content and reduced inhibitory α2-AR expression compared to males^(214)^. These sex-specific BAT differences convey an enhanced thermogenic potential and increased epinephrine-sensitivity to female animals. Consequently, it is crucial to include both sexes in studies aiming at modulators of non-shivering thermogenesis. In addition, various hormonal cues such as sex hormones (see phytoestrogens) or leptin affect BAT-mediated EE^(215,216)^. Surprisingly, circulating leptin levels are higher in male *v.* female rats, while this ratio is reciprocal in human subjects^(217,218)^. These examples substantiate two statements. First, energy homoeostasis is inherently modulated by sexual dimorphism and secondly, the underlying regulatory system is inconsistent between males and females across species. Sex as biological variable further affects the pharmacokinetics and metabolism of phytochemicals, especially with regards to their metabolisation by the gut microflora or liver^(219,220)^. As such, a key hepatic cytochrome P540 enzyme (CYP3A4) is elevated in females over males^(220)^ and CYP3A4 is predominantly metabolising BBR^(221)^, which could lead to sex-specific differences in the clearance of BBR. In the presented trials, the circulating metabolites are rarely determined and might not overlap between men and women causing differential study outcomes.

Finally, selected compounds clearly have a beneficial outcome in clinical trials and are accepted by meta-analyses. Regular catechin intake (300 mg) for example can result in about 418 kJ increase in EE. Spicy food hampers macronutrient intake by about 565 kJ and/or stabilises weight regain by compensating for the decrease in EE. This small contribution in itself might be sufficient to balance the energy gap to zero. Although these thermogenic effects are presently not sufficient to actively stimulate weight loss, they could be essential in preventing further fat accumulation and potentially stop the transition from overweight to obese states. As such, prevention of weight gain and weight maintenance are more easily achievable than a decrease in fat mass, as conserved mechanisms defend body weight rather than promote fat release^(222)^. In conclusion, dietary phytochemicals in their food matrix or as a supplement alone play an important role in obesity prevention by activating BAT functionality, targeting a broad population base.

## Supplementary material

For supplementary material accompanying this paper visit http://dx.doi.org/10.1017/S0029665120006928. click here to view supplementary material
