# Supplementary material for: Feeding brown fat: dietary phytochemicals targeting non-shivering thermogenesis to control body weight
Source: Proc Nutr Soc. 2020 Apr 15;79(3):338–56. doi: 10.1017/S0029665120006928 (PMC7663322; doi:10.1017/S0029665120006928)
Supplement: Supplementary file 1 [file S0029665120006928sup.zip › S0029665120006928sup001.pdf]

Table 1. Overview of rodent studies, clinical trials and *in vitro* assays addressing BAT-mediated thermogenesis by phytochemicals and their impact on energy expenditure and weight control.

Pterostilbene	blueberries, berries, wine, wine grapes, sorghum[1]			
Reference and model	Design	Sex and N	Treatment	Outcome
[2]<br>Golden Syrian Hamsters	DI	male<br>n=8-10 per group	2.5 mg/kg BW/d in HFD<br>3 weeks	-BW<br>plasma ↓C, ↓LDL, ↓LDL:HDL
[2]<br>rat hepatic H4IIEC3 cells		male	1,10,100 or 300 µM PTS<br>30 min	↑PPARα reporter activity
[3]<br>Zucker fa/fa rats	DI	male<br>n=10 per group	15 mg/kg BW/d in HFD<br>6 weeks	↓BW, ↓fat mass<br>plasma ↓C, ↓insulin
[4]<br>Wistar rats	DI	male<br>n=9 per group	15 or 30 mg/kg BW/d in HFHS<br>6 weeks	↓BW gain,<br>↓total fat mass, ↓vWAT, ↓sWAT<br>↑hepatic CPT1α and ACOX activity
[5]<br>Zucker fa/fa rats	orogastric catheter	male<br>n=10 per group	0, 15 or 30 mg/kg BW/d in chow diet,<br>6 weeks	↓BW, ↓AT weight<br>↑iBAT mRNA/protein: NRF1, UCP1, PPARα<br>↑iBAT CPT1b activity
[6]<br>OLETF rats	DI	male<br>n=6 per group	300 mg/kg BW/d in chow<br>4 weeks,	-BW, ↓abdominal WAT, ↓total WAT<br>↓RER, ↑FO, ↑EE<br>↓WAT FAS mRNA
[7]<br>C57BL/6 mice	DI	male and female<br>n=8 per group/sex	90 mg/kg BW/d in HFD<br>30 weeks	↓BW, ↑glucose tolerance (after 18 weeks)<br>↑iWAT thermogenic genes (PPARγ, PGC1α, SIRT1, CIDEA, TBX1), ↑iWAT UCP1 protein<br>➢ effects in m+f, stronger than in f vs m
[7] 3T3-L1 adipocytes	acute	male	5 µM PTS for 24 hours, d12	↑UCP1 protein, ↑CIDEA, FGF21 mRNA

[8]<br>hypercholesterolaemic<br>Caucasian, AA	placebo<br>parallel	male and female<br>100 mg/d, n=20<br>(m5/f15)<br>250 mg/d, n=20<br>(m6/f14)<br>placebo, n=20<br>(m7/f13)	100 or 250 mg/d<br>6-8 weeks	-BMI (if stratified for C medication ↓BMI)<br>plasma ↑total C, ↑LDL, -HDL
Resveratrol	peanuts and peanut products, grapes, red wine, soy, herbal remedies[9]			
[10]<br>C57Bl/6J mice	DI	male<br>n=8-10 per group	400 mg/kg BW/d in HFD<br>15 weeks	↓weight gain, ↓final BW, ↓WAT mass<br>↑VO_2, ↑cold-resistance (rectal T)<br>↑iBAT mito content, ↑iBAT thermogenic genes (UCP1, PGC1α, PPARα)<br>↑mito gene enrichment in muscle, ↑IS
[11]<br>C57BL/6NIA	DI	male<br>n=6-9 per group	22.4 mg/kg BW/d in HFD<br>middle aged mice,<br>for 55 weeks	-fat distribution, -BW (trend for↓), ↑IS, -BT<br>↑survival<br>plasma -TAG, ↑C, ↓GLC, ↓insulin
[12]<br>grey mouse lemur<br>(*Microcebus murinus*)	DI<br>Before-after	male<br>n=6	200 mg/kg BW/d<br>4 weeks	↑RER, ↓weight gain vs baseline week<br>↓food intake, -locomotor activity
[13]<br>mice (strain unknown)	DI	male<br>n=8 per group	0.4% w/w in chow diet<br>8 weeks	↓WAT mass, -BW, ↑VO_2, -locomotor activity<br>↑iBAT thermogenic genes (UCP1, PRDM16, SIRT1), plasma -C, -TAG, -GLC
[14]<br>CD-1 mice	DI	female<br>n=6 per group	0.1% w/w in HFD<br>4 weeks	↓BW gain, ↑VO_2, ↓RER, ↑EE (p=0.065),<br>↑UCP1+ in iWAT sections, ↓iWAT adipocyte size<br>↑pAMPK, UCP1, PRMD16 protein in iWAT<br>plasma ↓insulin, ↓TAG, -GLC

[14]<br>SVF from iWAT of CD-1 mice	long-term	unknown	10 µM RSV<br>during differentiation with brown adipogenic cocktail	↑thermogenic genes (UCP1, ELOVL3, PGC1α, CIDEA, PRDM16), ↑UCP1, PRDM16 protein<br>↑respiration, ↑pAMPK<br>➢ effects AMPK-dependent
[15]<br>CD-1 mice	DI	female	0.1% RSV w/w in HFD<br>4 weeks	↓BW gain, ↑BA number in iBAT sections<br>↑iBAT UCP1. PRDM16 and pAMPK protein
[16]<br>Spargue-Dawley rats	DI	male<br>n=8 per group	30 mg/kg BW/d in HFHS<br>6 weeks	-BW, ↓fat mass<br>↑BAT thermogenic genes (UCP1, PGC1α, TFAM)<br>↑iBAT UCP1 protein, ↓acetylated PGC1α (muscle)
[17]<br>obese, healthy (BMI>30)<br>Denmark	placebo<br>parallel	male<br>n=12 per group	500 mg RSV or placebo tablet/d<br>4 weeks	-BW, -total fat mass, -vWAT mass,<br>plasma –GLC, -C, -TAG,-ALT, -HbA1c<br>-acetylated lysine, pAMPK in muscle
[18]<br>non-obese, normoglycaemic<br>Caucasian	placebo<br>parallel	female<br>RSV n=15<br>placebo n=14	75 mg RSV/d<br>12 weeks	-BW, -fat mass, -sWAT mass, -vWAT mass<br>plasma -leptin,-C, -TAG, -NEFA, -GLC<br>-REE, -BP, -IS<br>-WAT microarray: -mito function, -FO genes<br>-muscle SIRT1 activity
[19]<br>older adults with IGT<br>US	before-after	male n=3<br>female n=7	1, 1.5 or 2 g RSV/d<br>4 weeks	-BW, -fat mass, -BP<br>plasma –C, -TAG, -insulin, -ALT, -insulin<br>↓post-meal GLC, ↓post-meal insulin
[20]<br>obese, healthy men	placebo<br>crossover	male n=11	150 mg RSV/d (resVida) or placebo,<br>30 days per treatment	-BW, -fat mass<br>↓SEE (caloric restriction), ↑diurnal RER<br>muscle ↑pAMPK, ↑mito activity, ↑SIRT1, PGC1α protein
Quercetin	apples, onions, black currants, red wine, black tea, nuts, seeds, shallots[21]			
[22]	DI	male	0.36% or 0.72% w/w OPE in	↓BW, ↓intra-abdominal fat mass

Sprague–Dawley rats		n=7 per group	HFD, 8 weeks	
[23]<br>Wistar rats	DI	male<br>n=12 per group	185, 270, 925 mg/kg BW/d in HFD<br>8 weeks	↓BW gain, ↓total fat mass, ↓vWAT<br>plasma ↓TAG, ↓NEFA, -C, ↓GLC at high dose<br>↓hepatic fat content, ↑fecal lipids<br>↑PPARα, ↑SIRT1, ↓ACC, ↓FAS mRNA (WAT)
[24]<br>Zucker fa/fa rats	oral gavage	male<br>n=7 per group	10 mg/kg BW/d RSV or vehicle with HFD<br>10 weeks	↓BW gain<br>plasma ↓TAG, ↓C, ↓GLC, ↓insulin, ↓HOMA-IR<br>↓TNFα production, iNOs protein in vWAT
[25]<br>C57BL/6	DI	male<br>n=6 for OPE<br>n=9 for control	0.5% w/w OPE in HFD<br>8 weeks	-BW gain, -eWAT mass, -rWAT mass<br>↑thermogenic genes (UCP1, PRDM16, CIDEA, PGC1α) in rWAT
[25]<br>3T3-L1 adipocytes	long-term	male	100 µM quercetin at d5, 7, 9	↑ UCP1, SIRT1, PGC1α mRNA and protein<br>↑pAMPK, pHSL, ↓lipogenic genes (FAS, ACC)
[26]<br>C57BL/6	DI	male<br>n=8-10 per group	0.1% w/w quercetin in HFD<br>8 weeks	-BW, -fat mass,<br>-EE, -RER, -FO, -CHO, ↓plasma TAG<br>↑sWAT thermogenic genes (UCP1, ELOVL3)<br>↑UCP1+ cells in sWAT, ↑FA uptake in sWAT<br>-mito content sWAT, -BAT morphology/genes
[27]<br>C57BL/6J	DI	male<br>n=8 per group	0.8% w/w in HFD<br>8 weeks	-BW, -adiposity, -EE, -RER<br>↓plasma inflammatory cytokines (INFγ, IL1, IL4)
[28]<br>C57BL/6	DI	male<br>n=6 per group	0.05% w/w quercetin in HFD<br>9 weeks	-BW, ↓WAT adipocyte size<br>↑UCP1+ cells, ↑UCP1, ↑PGC1α protein in iWAT<br>↑iWAT thermogenic genes (UCP1, PRDM16, MEM26, NRF-1)<br>↑PKA, pAMPK protein in iWAT<br>↑plasma NE, ↑iBAT UCP1

(29)<br>isolated Wistar rat adipocytes	acute	male	0, 1, 10, 100 or 250 µM quercetin, 15 min	↑PDE activity, ↑cAMP, ↑epinephrine-stimulated lipolysis
(30)<br>C57BL/6	DI	male<br>n=8 per group	0.1% w/w quercetin in HFD<br>12-17 weeks	↓BW, ↓sWAT mass, ↓eWAT mass, ↓eWAT cell size, plasma ↓leptin, ↓insulin, ↓TNFα, ↓IL6<br>↓mast cell infiltration eWAT<br>↓eWAT TNFα, IL6<br>↑eWAT SIRT1, pAMPK protein<br>↑iBAT UCP1 mRNA,
(31)<br>university students<br>healthy<br>Korean	placebo crossover	female<br>n=12	100 mg/d quercetin or placebo capsule for 2 weeks each	-BW, -fat mass, -WHR, -BMI, -SBP, -DBP<br>plasma –TAG, -C, -LDL
(32)<br>overweight/obese subjects (BMI>23 kg/m^2)<br>Korean	placebo crossover	female<br>quercetin n=18<br>placebo n=19	100 mg/d quercetin or placebo capsules<br>12 weeks	-BW, -fat mass, -BMI<br>plasma –ALT,-leptin, ↑adiponectin, -TNFα, -IL4
(33)<br>overweight/obese subjects<br>Korean	placebo crossover	male n=5<br>female n=31	100 mg/d quercetin or placebo capsules<br>12 weeks	-BW, -fat mas, -BMI, -WC, -RER, -REE<br>plasma –GLC, -C, -LDL, ↓TAG, -leptin<br>-before-after effects on REE, BW, RER, BMI within quercetin group
(34)<br>Meta-analysis	9 RCTs	male=189<br>female=336	100 to 1000 mg/d<br>2 to 12 weeks	-BW, -WC, -WHR, -BMI
Luteolin	peppers, carrots, cucumber, pomegranate, herbal spices, cabbage, broccoli, medicinal herbs (sage)[35]			
(36)<br>C57BL/6	DI	male<br>n=8 per group	0.01% w/w in HFD<br>12 weeks	↓BW, ↓sWAT mass, ↓vWAT mass, ↓BAT mass<br>↓eWAT adipocyte size, ↑IS<br>↓mast cell infiltration eWAT<br>plasma –insulin, ↓leptin, ↑adiponectin

(37)<br>C57BL/6	DI	male<br>n=13 per group	0.005% w/w in HFD<br>16 weeks	↓BW, ↓sWAT mass, ↓vWAT mass<br>plasma ↓TAG, ↓C, ↓NEFA, ↑IS<br>↑eWAT FAO genes (PGC1α, ADRB3, CPT2, PNP2, ACAD)
(38)<br>C57BL/6	DI	male<br>n=8 per group	0.01% w/w in HFD<br>20 weeks	↓BW, ↑IS<br>↓plasma MCP1, IL6, TNFα<br>↓macrophage infiltration vWAT, ↓M1/M2-ratio
(39)<br>C57BL/6	DI	male<br>n=12 per group	0.01% w/w in HFD<br>12 weeks	↓BW, ↓weight gain, ↓fat mass<br>↑VO_2, ↑CO2, ↑RER, ↑BAT UCP1 protein<br>↑UCP1+ cells in sWAT, ↑thermogenic genes (PGC1α, UCP1, SIRT1, PPARα, ELOVL3)<br>↑SIRT1, pAMPK, pACC protein in BAT, sWAT
(39)<br>primary subcutaneous and brown adipocytes	acute	unknown	24 hours of 100 nM luteolin on differentiated cells	↑SIRT1, UCP1, PGC1α protein<br>↑pAMPK, ↑pACC<br>↑thermogenic genes (UCP1, PRDM16, ELVOL6, PPARα)<br>➢ effects AMPK-dependent
Catechins	grapes, apples, strawberries, apricots, broad beans, cocoa-products, green/black/oolong tea[40,41]			
(42)<br>Sprague-Dawley rat	DI	male<br>n=8 per group	2% w/w green tea extract in HFD, 2 weeks	-BW, ↓fat mass, ↑BAT weight<br>↑BAT DNA/protein content<br>↑EE, propranolol prevented ↑EE
(43)<br>Sprague-Dawley rat	DI	male<br>n=8 per group	0.5% w/w catechins in chow<br>8 weeks	-BW<br>↑BAT mass, ↓pWAT mass, ↓eWAT mass<br>↑BAT UCP1 expression<br>plasma ↓TAG, GLC, leptin
(44)<br>New Zealand black mice	gavage<br>short-term	male<br>n=6 per group	3x 500 mg/kg EGCG or placebo, chow diet	-BW, -fat mass<br>-EE, ↓RER (p=0.053), -activity

[44]<br>New Zealand black mice	DI	male<br>n=11 per group	0.1% w/w EGCG in HFD<br>DIO 4 weeks, DI 4 weeks	↓BW, ↓fat mass, -food intake, ↓eWAT weight<br>plasma ↓TAG, -NEFA<br>-UCP1 mRNA in BAT
[45]<br>iBAT depots from Sprague-Dawely rat	acute	male	100 or 200 µM green tea extract for 40-90 min	↑iBAT respiration (100 µM)<br>↑norepinephrine (0.1 µM) stimulated respiration at 100 or 200 µM
[46]<br>healthy men<br>Geneva, CH	placebo crossover	male<br>n=10	3x daily capsule with 50mg caffeine and 80 mg EGCG, 50 mg caffeine or placebo	↑diurnal EE, ↑total EE, -nocturnal EE<br>↓total, diurnal and nocturnal RER<br>↑FO, ↑urinary norepinephrine excretion
[47]<br>young, healthy subjects<br>Lausanne, CH	placebo crossover	male n=15<br>female n=16	3x daily beverage with 100 mg caffeine and 180 mg catechins or placebo, 3 days	↑total EE, diurnal EE, nocturnal EE<br>-substrate oxidation<br>-catecholamine secretion
[48]<br>healthy men<br>(BMI 23-27 kg/m^2)<br>Laval University, CA	placebo crossover	male<br>n=14	3x daily capsule with 200 mg caffeine plus 90, 200, 300 or 400 mg EGCG or placebo	↑total EE, -SEE<br>-RER,-FO<br>-catecholamine secretion
[49]<br>overweight/obese men<br>(BMI=31 kg/m^2)<br>Berlin, DE	placebo crossover	male<br>n=10	300 or 600 mg EGCG or placebo capsule for 3 days	-EE (pre- and post-meal)<br>↓post-meal RQ, ↑post-meal FO (300 mg),<br>↓post-meal CHO (300 mg)<br>plasma -NEFA,-insulin, GLC
[50]<br>Meta-analysis<br>effect of EGCG on EE or anthropometric measures	8 RTC	n=268	EGCG:<br>300 or 600 mg/d for 2-3 days<br>300 to 800 mg/d for 2-12 weeks	↑EE, ↓RER, -FO, - BMI, ↓WC, -fat percentage
[51]<br>healthy men<br>Japanese	placebo crossover	male n=15<br>low BAT activity<br>(mean SUV=1.9)	615 mg catechins plus 77 mg caffeine or placebo (81 mg caffeine), 2x daily as beverage for 5 weeks	-BMI, -fat mass, -WC, -EE<br>↑cold-induced thermogenesis, ↑cold-induced FO

[51]<br>healthy men<br>Japanese	acute<br>crossover	male n=15	615 mg catechins plus 77 mg caffeine or placebo (81 mg caffeine), single beverage	↑post-drink EE,<br>↑EE in high BAT (SUV>2) vs low BAT subjects, pre-assessed by 2 hours cold-exposure
[52]<br>healthy university students<br>Japanese	placebo<br>parallel	female<br>catechin n=10<br>placebo n=11	640 mg catechins/d or placebo, beverage<br>12 weeks	-BMI, -fat mass, -BW<br>↑BAT density in supraclavicular region<br>neg. correlation between EMCL and BAT density
[53]<br>overweight/obese children<br>Japanese	placebo<br>parallel	catechin group (m21, f5)<br>placebo group (m13, f6)	576 mg/d catechins or placebo (75 mg/d catechins), as Oolong tea, 12weeks	-no changes in anthropometric or metabolites in catechin vs control<br>↓WC, ↓SBP, ↓LDLC in catechin group when stratified to baseline values
[54]<br>normal to overweight men<br>Japanese[36]	placebo<br>parallel	male<br>catechin group n=17<br>placebo group n=19	690 mg/d catechins or placebo (22 mg/d),<br>as Oolong tea, 12weeks	↓WC, ↓skinfold thickness, ↓total fat area<br>↓visWAT and sWAT area<br>plasma –NEFA, -TAG, -C, -GLC, -insulin
[55]<br>obese adult Thais<br>(BMI>25kg/m^2)	placebo<br>parallel	catechin group:<br>(m21, f9)<br>placebo group:<br>(m21, f9)	3x daily 250 mg catechins or placebo in capsule<br>12 weeks	↓BMI, ↓BW, ↓fat mass, ↓WC, -HC<br>↓RER, ↑REE
[56]<br>overweight/obese adults<br>(BMI >25-32 kg/m^2)<br>Caucasian	before-after	female n=63<br>male n=7	270 mg/d EGCG in capsule	-BW, ↓WC<br>-SBP, -DBP<br>plasma -C
[57]<br>Meta-analysis<br>effect of green tea extracts on anthropometry	15 RTCs	n=1243	catechin intake combined with caffeine intake (141 up to 1207 mg/d)<br>8 to 24 weeks	↓BMI, ↓BW, ↓WC, -WHR when compared to caffeine-intake only
Phytoestrogens	kidney beans, mung bean sprouts, Japanese arrowroot, soybean, soy products (tofu, soy milk, soy flour, soy sauce)[58]			

(59)<br>C57/B6J mice	DI	male<br>n=7-8	5% isoflavone-rich fraction of *Puerariae* flower in HFD,<br>7 weeks	↓BW, ↓WAT mass, ↓BAT mass<br>-food intake, -fecal lipid content<br>↑VO_2, -RER, ↑UCP1+ cells in BAT sections
(60)<br>CD-1 mice	DI	male n=12<br>female n=12	25% w/w soy-rich diet (150 pmm daidzein, 190 ppm genistein) vs soy-free diet<br>16 weeks	↓BW, ↓intra-abdominal fat mass, ↓iWAT, ↓eWAT/ovWAT, ↓WA adipocyte size<br>↓BAT mass (male), ↑brown appearance, ↓lipid droplet size<br>↑cold-resistance (rectal T), ↑VO_2, ↓RER (only male data available)
(61)<br>Sprague-Dawley rats	ovx<br>DI	female<br>n=10 per group	isoflavone-rich (200 µg/g) or isoflavone-free diet<br>13 days	↓BW gain, ↓abdominal fat mass<br>↓serum leptin
(62)<br>Long-Evans rats	DI	male	isoflavone-rich (600 ppm) or isoflavone-free (10-15 ppm) diet<br>up to 75 days of age	↑food intake, ↓BW gain, ↓WAT mass, ↓BAT mass<br>plasma ↑T3, ↓insulin, leptin<br>↑UCP1 protein in BAT
(63)<br>Wistar rats	DIO with<br>DI	male<br>n=16 per group	50 mg/kg BW daidzein or vehicle, i.p.<br>DIO 10 weeks, 14 d treatment	↓caloric intake, ↓BW gain<br>↓hepatic liver content<br>plasma ↓TAG, -C, ↑GLC, -ALT<br>↑UCP1 protein in BAT
(64)<br>ICR mice	DIO with gavage	male	0, 25, 50 or 100 mg/kg BW<br>DIO 8 weeks, 30 d treatment	↓BW. ↓vWAT mass, ↓sWAT mass<br>plasma ↓C, ↓LDL, ↓NEFA, -TAG, ↑HDL
(64)<br>primary adipocytes differentiated from eWAT SVF	acute	male	0, 1, 3, 16, 64 µM daidzein<br>24 hours	↑glycerol release (dose-dependent)

(65)<br>adipocytes from Wistar rats	acute	male	0.01, 0.1 or 1 mM daidzein	↑basal lipolysis (dose-dependent)<br>↑epinephrine-stimulated lipolysis (0.1 mM)<br>↓lipogenesis from GLC (0.1 and 1 mM)
(66)<br>C57BL/6	DI	female<br>n=8 per group	0.25% w/w genistein in HFD<br>8 weeks	↓BW, -sWAT weight, -vWAT weight<br>↓BAT weight (ns), ↑IS<br>plasma –TAG, -C, HDL-, ↓LDL, ↓NEFA<br>↑iWAT browning (UCP1, CIDEA mRNA)<br>↑ hypothalamic UNC3 mRNA
(67)<br>C57BL/6	DI	male<br>n=7-8 per group	0.2% w/w genistein in casein diet or casein only (control)<br>60 days	-BW, ↑glucose tolerance<br>↑thermogenic genes in sWAT (UCP1, PGC1α)<br>↑UCP1 protein in sWAT,-BAT<br>↑EE, ↑VO_2, ↑cold-resistance (rectal T), -RER<br>plasma –TAG, ↓GLC, ↓insulin
(67)<br>primary adipocytes from iWAT of mice	acute	unknown	0, 5, 15, 30 µM genistein for 1 hour	-basal respiration<br>↑maximal respiration
(68)<br>immortalized brown adipocytes	long-term	unknown	0, 0.1, 1 or 40 µM of genistein on differentiated adipocytes, 3 days treatment	↑UCP1 promoter activity (luciferase)<br>↑UCP1 activity (immunofluorescence intensity)
(69)<br>C57BL/6	oral gavage	male and female	50 to 200 mg/kg BW genistein or vehicle for 15 d	↓BAT mass, ↓eWAT (m), ↓abdominal WAT (f)<br>plasma ↓TAG, ↓C for 50 mg/kg BW
(70)<br>postmenopausal women (BMI=23.6 kg/m^2)<br>Chinese, equol-producer	placebo<br>parallel	female<br>n=90 per group	40 g soy flour, 40 g low-fat milk powder with 63 mg daidzein, 40 g low fat milk powder (placebo)<br>daily, 6 months	-BW, -BMI, -WC, -HC, -WHR, -fat mass

(71)<br>adolescent males<br>Tasmania	placebo<br>parallel	male<br>isoflavone n=69<br>placebo n=59	50 mg isoflavone equivalents or placebo tablets daily<br>6 weeks	-BW
(72)<br>obese women (20-65 yrs)<br>(BMI 30-40 kg/m^2)<br>USA	placebo<br>parallel	female<br>soy group n=22<br>casein group n=21	3x daily soy (50 mg isoflavone) or casein (3.5 mg isoflavone) shake,<br>16 weeks	-WC, -weight loss, -fat mass, -truncal fat<br>-SBP, -DBP
(73)<br>impaired glycemic control Chinese women (30-70 yrs)	placebo<br>parallel	female<br>daidzein n=55<br>genistein n=56<br>placebo n=54	10 g soy protein with no addition, 50 mg daidzein or 50 mg genistein<br>24 weeks	-BMI, -WC, -fat mass<br>-IS
(74)<br>patients with NAFLD<br>Iranian (16-69 yrs)	placebo<br>parallel	genistein group (m30, f11)<br>placebo group (m31, f10)	250 mg daidzein or placebo capsules<br>8 weeks	-BW, ↓fat percentage, ↓WHR, ↓WC, -BMI<br>plasma ↓TAG, -C, -LDL, -HDL, ↓insulin<br>↓HOMA-IR
(75)<br>postmenopausal women<br>(BMI>30 kg/m^2)<br>Caucasian or AA	placebo<br>parallel	soy group (n=17)<br>8 AA, 9 Caucasian<br>placebo (n=16)<br>8 AA, 8 Caucasian	soy protein with isoflavones (160 mg) or placebo casein, shake<br>3 months	-BW, -total fat, -lean mass<br>↓abdominal, ↓subcutaneous abdominal fat, ↓vWAT<br>for AA: weight loss more than for Caucasian<br>for Caucasian: vWAT loss bigger than for AA<br>plasma ↓IL6, -CRP, -TNFα, -leptin, -HDL, -LDL, -C, -TAG
(76)<br>postmenopausal women<br>(mean BMI=30.5)<br>Caucasian	placebo<br>parallel	female<br>soy group n=9<br>placebo n=6	soy protein with isoflavones (160 mg) or placebo casein, shake<br>3 months	-BMI, -BW, -total fat mass, -IS<br>↓subcutaneous abdominal fat, ↓intra-abdominal fat<br>plasma -GLC, -insulin

[77]<br>Meta-analysis<br>Effect of soy-isoflavones on BW in non-Asian, postmenopausal women	9 RCTs	isoflavones n=272<br>placebo n=256	40 to 160 mg/d of isoflavones<br>8 weeks to 1 year	↓BW with isoflavone intake<br><100 mg or <6 months more effective<br>more effective with BMI<30 kg/m^2
[78]<br>Meta-analysis<br>effect of soy and isoflavones on anthropometric measures	24 soy RTCs<br>17 isoflavones RTCs	soy:<br>f1265,<br>m45 (1 RTC)<br>m/f =74 (mixed)<br><br>isoflavones:<br>f1177, m0	soy protein: 7.5 to 116 mg/d<br>4 weeks to 2 years<br>isoflavones: 33.3 to 300 mg/d<br>8 weeks to 2 years	soy:<br>-BW, >40 g/d ↑BW, 1-3 months ↑weight gain<br>-WC, -fat mass<br>isoflavones:<br>↓BMI for postmenopausal and Caucasian women<br><100 mg and 2-6 months more effective<br>-fat mass, -WC
Capsaicinoids	chili, bell peppers, jalapenos, habaneros, cayenne pepper, red pepper[79,80]			
[81]<br>Std ddY mice	intragastric tube	unknown<br>n=6-8	vehicle, 10 mg/kg BW capsaicin or 10, 50 mg/kg BW capsiate, 2 weeks	↓BW (ns), -food intake<br>-BAT mass, ↓eWAT for capsiate, ↓pWAT for capsaicin and 50 mg/kg capsiate
[81]<br>Std ddY mice	intragastric tube, acute	unknown<br>n=6-8	Vehicle, 10 mg/kg BW capsaicin or 10 mg/kg BW capsiate, 3 hours	↑VO_2 for capsaicin and capsiate,<br>↑serum adrenaline<br>plasma ↑NEFA, ↓TAG
[82]<br>C57BL/6 or TRPV1 -/- mice	intragastric tube, acute	male<br>n=5-18	vehicle, 10 mg/kg BW capsaicin or 10 mg/kg BW capsiate, 3 hours	↑VO_2 (capsaicin, capsiate at 10 mg/kg BW)<br>↑FO (capsaicin, capsiate at 10 mg/kg BW), ↓CHO<br>↑BAT and colonic T (50 mg/kg capsinoids, 10 mg/kg capsaicin)<br>↓T increase after denervation of jejunal nerves at 50 mg/kg capsinoid<br>- effects in wt but not TRPV-/- mice
[83]<br>TRPV1 -/- or wt mice	DI	male	0.01% w/w capsaicin in HFD<br>32 weeks	↓weight gain, ↓BW,<br>↑BAT UCP1, BMP8b protein

B6.129X1				↑activity, ↑RER, -food intake,<br>↓BAT TAG content, ↑BAT glycerol release (basal or forskolin-stimulated)<br>↑TRPV1 protein in BAT<br>↑Ca^{2+} influx in isolated BA (2 µM CAP)<br>↑pAMPK, pSIRT1 in BAT<br>↓PRDM16, PPARγ acetylation (HEK293 1 µM CAP), ↑PRDM16 and PPARγ interaction in BAT lysate<br>➢ effects blunted in TRPV-/- vs wt mice
(84)<br>TRPV1 -/- or wt mice<br>B6.129X1	DI	male<br>n=40 per group	0.01% w/w capsaicin in HFD<br>26 weeks	↓weight gain,<br>↑TRPV1 mRNA in iWAT, eWAT<br>↑EE, ↑activity, -fecal lipid content, ↑RER, ↑VO_2<br>↑UCP1, BMP8b, PPARα/γ protein in s/eWAT,<br>↑sWAT lipolysis (basal, forskolin-stimulated)<br>↓PRDM16, PPARγ acetylation in sWAT<br>↑pAMPk, CaMKKII activation in sWAT<br>↑Ca^{2+} influx in isolated WA (2 µM CAP)<br>➢ effects blunted in TRPV-/- vs wt mice
(85)<br>C57BL/6	DIO+DI	male<br>n=6 per group	0.01% w/w capsaicin in HFD<br>DIO 10 weeks, 10 week DI	↓BW, ↓weight gain, ↑eWAT, ↓rWAT mass<br>-food intake, ↓WAT adipocyte size<br>↑glucose tolerance, ↑adiponectin, ↓leptin<br>↑TRPV1 expression WAT
(86)<br>Sprague-Dawley rats	intra-muscular	female<br>n=9-18 per group	0.6, 0.7, 0.8 mg/kg BW capsaicin or DMSO, 80 to 120 min	↑BAT and rectal T with 0.8 mg/kg BW<br>-BAT weight, -mito content<br>↑BAT respiration
(87)<br>Std ddY mice	intragastric tube	male<br>n=9-10	10 mg/kg BW capsiate or vehicle,<br>2 weeks	-BAT mass, ↓eWAT, ↓pWAT<br>↑VO_2, ↑FO, ↑CHO, ↑UCP1 protein (BAT)<br>↑UCP1 mRNA (eWAT, BAT),

(87)<br>Std ddY mice	intragastric tube, acute	male<br>n=4 per group	10 mg/kg BW capsiate or vehicle for 30 min	↑UCP1 mRNA in BAT
(88)<br>healthy young men<br>British	acute	male	breakfast with 3g chili sauce	↑post-meal EE
(89)<br>long distance runners<br>Japanese	acute<br>crossover	male<br>n=8	breakfast with or without 10 g red pepper	↑post-meal EE (30 min)<br>↑RER, ↑CHO, ↓FO<br>➢ effect blocked by propranolol
(90)<br>healthy young men<br>Caucasian	placebo<br>crossover	male<br>n=10	appetizer with or without 6 g of red pepper	↓energy intake at lunch and dinner<br>↑sympathetic: parasympathetic nerve activity
(91)<br>healthy lean subjects<br>Caucasian	placebo<br>crossover	male n=11<br>female n=19	1030 mg red pepper in lunch	-EE post-meal<br>-RER<br>-CHO, -FO, ↑peak plasma GLP-1,-ghrelin
(92)<br>overweight subjects<br>(mean BMI=29.4 kg/m^2)<br>Caucasian, AA, other	placebo<br>parallel	male (ethnicity)<br>Placebo (16,9,3)<br>3 mg (15, 9, 1)<br>9 mg (10, 4, 2)	0, 3, 9 mg dihydrocapsiate in gel capsule<br>4 weeks	-RMR (p=0.054 for 3 mg vs placebo)<br>↑RMR dihydrocapsiate vs placebo
(93)<br>Meta-analysis<br>effect of capsaicin or capsiate on EE, RER	capsiate on EE 13 RCTs<br>capsiate on RER 9 RCTs<br>capsaicin on EE 13 RCTs<br>capsaicin on RER 10 RCTs<br>female/male unknown		capsaicin doses: <7, 20-35 mg, 135-150 mg/d<br>dihydrocapsiate doses: <1.5, 2-4 or 6-9 mg/d	↑EE at 2-9 mg/d for capsiate<br>↑EE at 135-150 mg/d for capsaicin<br>↑FO at 6-9 mg/d capsiate<br>↑FO at 20-150 mg/d for capsaicin<br>↑SNS activity (1 RCT)
(94)<br>healthy adult participants<br>(BMI 20-30)<br>Caucasian	acute<br>controlled	N=15 per group<br>(m8, f7)	0 or 7.68 mg/d capsaicin with 100% or 75% of daily energy requirements<br>26 hours	-TEE 100% CAP vs 100% control (c)<br>-DIT 75% CAP vs 100% c, ↓75 % c vs 100%<br>-REE 75% CAP vs 100% c, ↓75 % c vs 100%<br>↑FAO 75% CAP vs 100% c, -75 % c vs 100%<br>↓CHO 75% CAP vs 100% c

(95)<br>healthy adults<br>BMI 25-30 kg/m^2<br>44% Hispanic, 41% white, non-Hispanic, 13% black, 2% others	placebo<br>parallel	capsinoids<br>(m21, f22)<br>Placebo<br>(m17, f20)	6 mg capsinoids or placebo capsule with MCTG, rapeseed oil<br>12 weeks	-BW, -fat mass, -abdominal fat<br>-EE (only m): 54 kcal/d higher with CAP (p=019)<br>-FO (only m): 21 mg/min higher with CAP (p=0.06)
(96)<br>lean, healthy subjects<br>Singapore	crossover<br>cold vs capsinoids	Male n=8,<br>female n=12<br>BAT+ m6/f6<br>BAT- m2/f6	12 mg capsinoids in capsule with rapeseed oil and MCTG	↑FDG-uptake in BAT<br>↑EE, higher in BAT+ subjects<br>↑FO, ↓RER<br>plasma –GLC, -TAG, ↑C, ↑NEFA
(97)<br>young healthy men<br>Japanese	placebo<br>crossover<br>acute	male n=18<br>BAT+ n=10<br>BAT- n=8	9 mg capsinoids or placebo capsule with rapeseed oil, MCTG<br>2 hours	↑EE in BAT+ with capsinoids vs placebo<br>-RER, -skin T
Berberine	barberry, supplements from bark, root, stems or leaves from plants of the *Berberis* genus (e.g. goldenseal, goldthread, Oregon grape, tree turmeric)[98]			
(99)<br>db/db mice	i.p.	male<br>BBR n=17<br>vehicle n=16	5 mg/kg BW on chow diet<br>26 days	↓BW, ↓eWAT mass, ↓sWAT adipocyte size<br>↓intra-abdominal fat, ↑IS,<br>↑BAT mRNA, PPARα, PGC1α ↓FAS<br>↓WAT mRNA FAS, PPARγ, SREBP1c, aP2
(99)<br>3T3-L1 adipocytes	acute	male	5 µg/mL BBR for 60 min	↑pAMPK, ↑pACC
(100)<br>db/db mice	long-term<br>i.p	male<br>n=5 per group	5 mg/kg BW on chow diet<br>26 days at 22°C or 30°C	↓BW, ↓fat mass, ↓plasma NEFA<br>↑rectal T, ↑VCO_2, ↑VO_2, ↑EE<br>↑cold-resistance (core T)<br>↑BAT activity (PET/CT)<br>↑BAT mito content, ↑oxphos, ↓BAT mass<br>↑BAT UCP1, PGC1α, CPT1, pAMPK protein

				↑iWAT thermogenic genes (UCP1, NRF1)<br>↑UCP1+ cells, ↑mito content, ↑UCP1 protein<br>➢ effects blunted at 30°C
(101)<br>C57BL/6	DI	male	5 mg/kg BW, i.p.<br>on chow diet, 4 weeks	↑hepatic FGF21 expression, ↑plasma FGF21<br>↑iBAT mRNA (UCP1, DIO2, PRMD16)
(102)<br>C57BL/6	DIO	male<br>n=7-8	1.5 mg/kg BW/d on HFD<br>DIO 8 weeks, DI 6 weeks	↓BW gain, ↓pWAT, iWAT mass<br>↑rectal T, ↑EE, ↑VO_2, ↑BAT activity, volume (PET/CT), ↑UCP1+ cells in BAT<br>↓BAT PRDM16 promoter methylation<br>➢ effects blunted in adiponectinCre AMPKα1/2 mice
(102)<br>BAT SVF cells	long-term	male	250 nM BBR<br>during differentiation until d8	↑basal and uncoupled respiration<br>↑BA adipogenesis (↑UCP1+ cells)<br>↑fatty acid oxidative BAT-specific genes<br>↑UCP1, PGC1α, PRDM16 protein
(102)<br>patients with NAFLD<br>mean BMI=29 kg/m2<br>China	before-after	not defined	1.5 g BBR/d<br>1 month	↓BW, ↓WC, ↓BMI, -total fat mass,<br>↓vWAT mass, ↓sWAT mass<br>↓HOMA-IR<br>↑BAT volume, BAT activity
(103)<br>subjects with NAFLD<br>China	controlled parallel	LSI (m32/f30)<br>LSI+P (m28/f32)<br>LSI+BBR (m38/f24)	LSI+1.5g BBR/d, LSI+15 mg/d pioglitazone, LSI only<br>16 weeks	↓hepatic fat content vs LSI,<br>↓BW, ↓BMI vs LSI and vs LSI+pioglitazone<br>-HbA1c, ↓HOMA-IR vs LSI,
(104)<br>newly diagnosed diabetics<br>no pharmacotherapy<br>China	placebo parallel	placebo (m38, f28)<br>BBR (m31, f21)	1.5 g BBR/d or placebo<br>12 weeks	-BW, ↓BMI, -WHR<br>plasma –FBG, ↓PBG, ↓HbA1c, -HOMA-IR, ↓C,<br>- insulin, -TAG

(105)<br>type 2 diabetics with poor glycemic control<br>China	before-after	n=48<br>sex not stated	1.5 g BBR/d plus prescribed diabetes medication<br>12 weeks	↓WC, ↓WHR, -BMI<br>plasma ↓FBG, ↓PBG, -TAG, ↓C, ↓insulin<br>↓HOMA-IR
(106)<br>obese adults<br>Caucasian	before-after	male n=2<br>female n=5	1.5 g BBR/d<br>12 weeks	-BMI, -WHR, - fat percentage<br>plasma –GLC,-TAG, ↓C, ↓ALT, ↓AST<br>-plasma inflammatory markers

AA, African American, BAT, brown adipose tissue; BBR, berberine; BW, body weight; BMI, body mass index, C, cholesterol; CHO, carbohydrate oxidation; d, day; DI, dietary intervention; DIO, diet-induced obesity; DIT, diet-induced thermogenesis; EE, energy expenditure; EMCL, extramyocellular lipid; f, female; FBG, fasting blood glucose; FO, fat oxidation; HC, hip circumference; HFHS, high fat high sucrose; IS, insulin sensitivity, IGT, impaired glucose tolerance; i.p., intraperitoneal; LSI, life style intervention; m, male; MCTG, medium chain triglyceride; mito, mitochondria(l); NAFLD, non-alcoholic fatty liver disease, NEFA, non-esterified fatty acids; ovx, ovariectomized, PBG, postprandial blood glucose; RCT, randomized controlled trial; REE, resting energy expenditure; RER, resting energy expenditure; SEE, sleeping energy expenditure; SUV, standardized uptake value; T, temperature; TAG, triacylglycerol, TEE, total energy expenditure; WC, waist circumference; WHR, waist-hip-ratio; WAT, white adipose tissue, wt, wild-type; - unchanged, ↓decrease, ↑increase compared to placebo or baseline

References

1. Reinisalo M, Kårlund A, Koskela A, *et al.* (2015) Polyphenol Stilbenes: Molecular Mechanisms of Defence against Oxidative Stress and Aging-Related Diseases. *Oxid Med Cell Longev* **2015**, 340520.

2. Rimando AM, Nagmani R, Feller DR, *et al.* (2005) Pterostilbene, a new agonist for the peroxisome proliferator-activated receptor alpha-isoform, lowers plasma lipoproteins and cholesterol in hypercholesterolemic hamsters. *J Agric Food Chem* **53**, 3403–3407.

3. Etxeberria U, Hijona E, Aguirre L, *et al.* (2017) Pterostilbene-induced changes in gut microbiota composition in relation to obesity. *Mol Nutr Food Res* **61**, 1500906

4. Gómez-Zorita S, Fernández-Quintela A, Lasa A, *et al.* (2014) Pterostilbene, a dimethyl ether derivative of resveratrol, reduces fat accumulation in rats fed an obesogenic diet. *J Agric Food Chem* **62**, 8371–8378.

5. Aguirre L, Milton-Laskibar I, Hijona E, *et al.* (2016) Effects of pterostilbene in brown adipose tissue from obese rats. *J Physiol Biochem* **73**, 457–464.

6. Nagao K, Jinnouchi T, Kai S, *et al.* (2017) Pterostilbene, a dimethylated analog of resveratrol, promotes energy metabolism in obese rats. *J Nutr Biochem* **43**, 151–155.

7. La Spina M, Galletta E, Azzolini M, *et al.* (2019) Browning Effects of a Chronic Pterostilbene Supplementation in Mice Fed a High-Fat Diet. *Int J Mol Sci* **20**, 5377.

8. Riche DM, Riche KD, Blackshear CT, *et al.* (2014) Pterostilbene on metabolic parameters: a randomized, double-blind, and placebo-controlled trial. *Evid.-Based Complement Alter Med ECAM* ***2014***, 459165.

9. Burns J, Yokota T, Ashihara H, *et al.* (2002) Plant foods and herbal sources of resveratrol. *J Agric Food Chem* **50**, 3337–3340.

10. Lagouge M, Argmann C, Gerhart-Hines Z, *et al.* (2006) Resveratrol improves mitochondrial function and protects against metabolic disease by activating SIRT1 and PGC-1alpha. *Cell* **127**, 1109–1122.

11. Baur JA, Pearson KJ, Price NL, *et al.* (2006) Resveratrol improves health and survival of mice on a high-calorie diet. *Nature* **444**, 337–342.

12. Dal-Pan A, Blanc S & Aujard F (2010) Resveratrol suppresses body mass gain in a seasonal non-human primate model of obesity. *BMC Physiol* **10**, 11.

13. Andrade JMO, Frade ACM, Guimarães JB, *et al.* (2014) Resveratrol increases brown adipose tissue thermogenesis markers by increasing SIRT1 and energy expenditure and decreasing fat accumulation in adipose tissue of mice fed a standard diet. *Eur J Nutr* **53**, 1503–1510.

14. Wang S, Liang X, Yang Q, *et al.* (2015) Resveratrol induces brown-like adipocyte formation in white fat through activation of AMP-activated protein kinase (AMPK) α1. *Int J Obes 2005* **39**, 967–976.

15. Wang S, Liang X, Yang Q, *et al.* (2017) Resveratrol enhances brown adipocyte formation and function by activating AMP-activated protein kinase (AMPK) α1 in mice fed high-fat diet. *Mol Nut Food Res* **61**, 1600746.

16. Alberdi G, Rodríguez VM, Miranda J, *et al.* (2013) Thermogenesis is involved in the body-fat lowering effects of resveratrol in rats. *Food Chem* **141**, 1530–1535.

17. Poulsen MM, Vestergaard PF, Clasen BF, *et al.* (2013) High-Dose Resveratrol Supplementation in Obese Men. *Diabetes* **62**, 1186–1195.

18. Yoshino J, Conte C, Fontana L, *et al.* (2012) Resveratrol supplementation does not improve metabolic function in nonobese women with normal glucose tolerance. *Cell Metab* **16**, 658–664.

19. Crandall JP, Oram V, Trandafirescu G, *et al.* (2012) Pilot study of resveratrol in older adults with impaired glucose tolerance. *J Gerontol A Biol Sci Med Sci* **67**, 1307–1312.

20. Timmers S, Konings E, Bilet L, *et al.* (2011) Calorie restriction-like effects of 30 days of Resveratrol (resVida^{TM}) supplementation on energy metabolism and metabolic profile in obese humans. *Cell Metab* **14,** 612-622.

21. Li Y, Yao J, Han C, *et al.* (2016) Quercetin, Inflammation and Immunity. *Nutrients* **8**, 187

22. Moon J, Do H-J, Kim OY, *et al.* (2013) Antiobesity effects of quercetin-rich onion peel extract on the differentiation of 3T3-L1 preadipocytes and the adipogenesis in high fat-fed rats. *Food Chem Toxicol Int J Publ Br Ind Biol Res Asso* **58**, 347–354.

23. Ting Y, Chang W-T, Shiau D-K, *et al.* (2018) Antiobesity Efficacy of Quercetin-Rich Supplement on Diet-Induced Obese Rats: Effects on Body Composition, Serum Lipid Profile, and Gene Expression. *J. Agric Food Chem* **66**, 70–80.

24. Rivera L, Morón R, Sánchez M, *et al.* (2008) Quercetin ameliorates metabolic syndrome and improves the inflammatory status in obese Zucker rats. *Obes. Silver Spring Md* **16**, 2081–2087.

25. Lee SG, Parks JS & Kang HW (2017) Quercetin, a functional compound of onion peel, remodels white adipocytes to brown-like adipocytes. *J Nutr Biochem* **42**, 62–71.

26. Kuipers EN, van Dam AD, Held NM, *et al.* (2018) Quercetin Lowers Plasma Triglycerides Accompanied by White Adipose Tissue Browning in Diet-Induced Obese Mice. *Int J Mol Sci* **19**.

27. Stewart LK, Soileau JL, Ribnicky D, *et al.* (2008) Quercetin transiently increases energy expenditure but persistently decreases circulating markers of inflammation in C57BL/6J mice fed a high-fat diet. *Metabolism* **57**, S39–S46.

28. Choi H, Kim C-S & Yu R (2018) Quercetin Upregulates Uncoupling Protein 1 in White/Brown Adipose Tissues through Sympathetic Stimulation. *J Obes Metab Syndr* **27**, 102–109.

29. Kuppusamy UR & Das NP (1994) Potentiation of beta-adrenoceptor agonist-mediated lipolysis by quercetin and fisetin in isolated rat adipocytes. *Biochem Pharmacol* **47**, 521–529.

30. Dong J, Zhang X, Zhang L, *et al.* (2014) Quercetin reduces obesity-associated ATM infiltration and inflammation in mice: a mechanism including AMPKα1/SIRT1. *J Lipid Res* **55**, 363–374.

31. Kim J, Cha Y-J, Lee K-H, *et al*. (2013) Effect of onion peel extract supplementation on the lipid profile and antioxidative status of healthy young women: a randomized, placebo-controlled, double-blind, crossover trial. *Nutr Res Pract* **7**, 373–379.

32. Kim K-A & Yim J-E (2016) The Effect of Onion Peel Extract on Inflammatory Mediators in Korean Overweight and Obese Women. *Clin Nutr Res* **5**, 261–269.

33. Lee J-S, Cha Y-J, Lee K-H, *et al.* (2016) Onion peel extract reduces the percentage of body fat in overweight and obese subjects: a 12-week, randomized, double-blind, placebo-controlled study. *Nut Res Pract* **10**, 175–181.

34. Huang H, Liao D, Dong Y, *et al*. (2019) Clinical effectiveness of quercetin supplementation in the management of weight loss: a pooled analysis of randomized controlled trials. *Diabetes Metab Syndr Obes Targets Ther* **12**, 553–563

35. López-Lázaro M (2009) Distribution and biological activities of the flavonoid luteolin. *Mini Rev Med Chem* **9**, 31-59

36. Xu N, Zhang L, Dong J, *et al.* (2014) Low-dose diet supplement of a natural flavonoid, luteolin, ameliorates diet-induced obesity and insulin resistance in mice. *Mol Nutr Food Res* **58**, 1258–1268.

37. Kwon E-Y, Jung UJ, Park T, *et al*. (2015) Luteolin attenuates hepatic steatosis and insulin resistance through the interplay between the liver and adipose tissue in mice with diet-induced obesity. *Diabetes* **64**, 1658–1669.

38. Zhang L, Han Y-J, Zhang X, *et al*. (2016) Luteolin reduces obesity-associated insulin resistance in mice by activating AMPKα1 signalling in adipose tissue macrophages. *Diabetologia* **59**, 2219–2228.

39. Zhang X, Zhang Q-X, Wang X, *et* al (2016) Dietary luteolin activates browning and thermogenesis in mice through an AMPK/PGC1α pathway-mediated mechanism. *Int J Obes 2005* **40**, 1841–1849.

40. Henning SM, Fajardo-Lira C, Lee HW, et al. (2003) Catechin Content of 18 Teas and a Green Tea Extract Supplement Correlates With the Antioxidant Capacity. *Nutr Cancer* **45**, 226–235.

41. Arts ICW, van de Putte B & Hollman PCH (2000) Catechin Contents of Foods Commonly Consumed in The Netherlands. 2. Tea, Wine, Fruit Juices, and Chocolate Milk. *J. Agric Food Chem.* **48**, 1752–1757.

42. Choo JJ (2003) Green tea reduces body fat accretion caused by high-fat diet in rats through beta-adrenoceptor activation of thermogenesis in brown adipose tissue. *J. Nutr Biochem* **14**, 671–676.

43. Nomura S, Ichinose T, Jinde M, *et al*. (2008) Tea catechins enhance the mRNA expression of uncoupling protein 1 in rat brown adipose tissue. *J Nut. Biochem* **19**, 840–847.

44. Klaus S, Pültz S, Thöne-Reineke C, *et al*. (2005) Epigallocatechin gallate attenuates diet-induced obesity in mice by decreasing energy absorption and increasing fat oxidation. *Int J Obes 2005* **29**, 615–623.

45. Dulloo AG, Seydoux J, Girardier L, *et al.* (2000) Green tea and thermogenesis: interactions between catechin-polyphenols, caffeine and sympathetic activity. *Int J Obes Relat Metab Disord J Int Assoc Study Obes* **24**, 252–258.

46. Dulloo AG, Duret C, Rohrer D, *et al*. (1999) Efficacy of a green tea extract rich in catechin polyphenols and caffeine in increasing 24-h energy expenditure and fat oxidation in humans. *Am. J. Clin. Nutr* **70**, 1040–1045.

47. Rudelle S, Ferruzzi MG, Cristiani I, *et al*. (2007) Effect of a thermogenic beverage on 24-hour energy metabolism in humans. *Obes Silver Spring Md* **15**, 349–355.

48. Bérubé-Parent S, Pelletier C, Doré J, *et al.* (2005) Effects of encapsulated green tea and Guarana extracts containing a mixture of epigallocatechin-3-gallate and caffeine on 24 h energy expenditure and fat oxidation in men. *Br J Nutr* **94**, 432–436.

49. Thielecke F, Rahn G, Böhnke J, *et al.* (2010) Epigallocatechin-3-gallate and postprandial fat oxidation in overweight/obese male volunteers: a pilot study. *Eur J Clin Nutr* **64**, 704–713.

50. Kapoor MP, Sugita M, Fukuzawa Y, *et al.* (2017) Physiological effects of epigallocatechin-3-gallate (EGCG) on energy expenditure for prospective fat oxidation in humans: A systematic review and meta-analysis. *Nutr Biochem* **43**, 1–10.

51. Yoneshiro T, Matsushita M, Hibi M, *et al.* (2017) Tea catechin and caffeine activate brown adipose tissue and increase cold-induced thermogenic capacity in humans. *Am. J Clin Nutr* **105**, 873–881.

52. Nirengi S, Amagasa S, Homma T, *et al*. (2016) Daily ingestion of catechin-rich beverage increases brown adipose tissue density and decreases extramyocellular lipids in healthy young women. *SpringerPlus* **5**, 1363.

53. Matsuyama T, Tanaka Y, Kamimaki I, *et al*. (2008) Catechin safely improved higher levels of fatness, blood pressure, and cholesterol in children. *Obes Silver Spring Md* **16**, 1338–1348.

54. Nagao T, Komine Y, Soga S, *et al*. (2005) Ingestion of a tea rich in catechins leads to a reduction in body fat and malondialdehyde-modified LDL in men. *Am J Clin Nut.* **81**, 122–129.

55. Auvichayapat P, Prapochanung M, Tunkamnerdthai O, *et al.* (2008) Effectiveness of green tea on weight reduction in obese Thais: A randomized, controlled trial. *Physiol Behav* **93**, 486–491.

56. Chantre P & Lairon D (2002) Recent findings of green tea extract AR25 (Exolise) and its activity for the treatment of obesity. *Phytomedicine* **9**, 3–8.

57. Phung OJ, Baker WL, Matthews LJ, *et al.* (2010) Effect of green tea catechins with or without caffeine on anthropometric measures: a systematic review and meta-analysis. *Am J Clin Nutr* **91**, 73–81.

58. Zaheer K & Humayoun Akhtar M (2017) An updated review of dietary isoflavones: Nutrition, processing, bioavailability and impacts on human health. *Crit Rev Food Sci Nutr* **57**, 1280–1293.

59. Kamiya T, Nagamine R, Sameshima-Kamiya M, *et al.* (2012) The isoflavone-rich fraction of the crude extract of the Puerariae flower increases oxygen consumption and BAT UCP1 expression in high-fat diet-fed mice. *Glob J Health Sc.* **4**, 147–155.

60. Cederroth CR, Vinciguerra M, Kühne F, *et al.* (2007) A Phytoestrogen-Rich Diet Increases Energy Expenditure and Decreases Adiposity in Mice. *Environ Health Perspect* **115**, 1467–1473.

61. Russell AL, Grimes JM, Cruthirds DF, *et al.* (2017) Dietary Isoflavone-Dependent and Estradiol Replacement Effects on Body Weight in the Ovariectomized (OVX) Rat. *Horm Metab Res Horm Stoffwechselforschung Horm Metab* **49**, 457–465.

62. Lephart ED, Porter JP, Lund TD, *et al.* (2004) Dietary isoflavones alter regulatory behaviors, metabolic hormones and neuroendocrine function in Long-Evans male rats. *Nutr. Metab.* **1**, 16.

63. Crespillo A, Alonso M, Vida M, *et al.* (2011) Reduction of body weight, liver steatosis and expression of stearoyl-CoA desaturase 1 by the isoflavone daidzein in diet-induced obesity. *Br J Pharmacol* **164**, 1899–1915

64. Guo Y, Wu G, Su X, *et al.* (2009) Antiobesity action of a daidzein derivative on male obese mice induced by a high-fat diet. *Nutr Res N. Y. N* **29**, 656–663.

65. Szkudelska K, Szkudelski T & Nogowski L (2002) Daidzein, coumestrol and zearalenone affect lipogenesis and lipolysis in rat adipocytes. *Phytomedicine Int J Phytother Phytopharm* **9**, 338–345.

66. Zhou L, Xiao X, Zhang Q, *et al.* (2019) A Possible Mechanism: Genistein Improves Metabolism and Induces White Fat Browning Through Modulating Hypothalamic Expression of Ucn3, Depp, and Stc1. *Front Endocrino.* **10**, 478.

67. Palacios-González B, Vargas-Castillo A, Velázquez-Villegas LA, *et al.* (2019) Genistein increases the thermogenic program of subcutaneous WAT and increases energy expenditure in mice. *J Nutr Biochem* **68**, 59–68.

68. Buhlmann E, Horváth C, Houriet J, *et al.* (2019) Puerariae lobatae root extracts and the regulation of brown fat activity. *Phytomedicine Int J Phytother Phytopharm* **64**, 153075.

69. Penza M, Montani C, Romani A, *et al.* (2006) Genistein affects adipose tissue deposition in a dose-dependent and gender-specific manner. *Endocrinology* **147**, 5740–5751.

70. Liu Z-M, Ho SC, Chen Y-M, *et al.* (2013) A six-month randomized controlled trial of whole soy and isoflavones daidzein on body composition in equol-producing postmenopausal women with prehypertension. *J Obes* **2013**, 359763.

71. Jones G, Dwyer T, Hynes K, *et al*. (2003) A randomized controlled trial of phytoestrogen supplementation, growth and bone turnover in adolescent males. *Eur J Clin Nut.* **57**, 324–327.

72. Anderson JW, Fuller J, Patterson K, *et al.* (2007) Soy compared to casein meal replacement shakes with energy-restricted diets for obese women: randomized controlled trial. *Metabolism* **56**, 280–288.

73. Ye Y-B, Chen A-L, Lu W, *et al*. (2015) Daidzein and genistein fail to improve glycemic control and insulin sensitivity in Chinese women with impaired glucose regulation: A double-blind, randomized, placebo-controlled trial. *Mol Nut Foodes* **59**, 240–249.

74. Amanat S, Eftekhari MH, Fararouei M, *et al.* (2018) Genistein supplementation improves insulin resistance and inflammatory state in non-alcoholic fatty liver patients: A randomized, controlled trial. *Clin Nutr Edinb Scotl* **37**, 1210–1215.

75. Christie DR, Grant J, Darnell BE, *et al*. (2010) Metabolic effects of soy supplementation in postmenopausal Caucasian and African American women: a randomized, placebo-controlled trial. *Am J Obstet Gynecol* **203**, 153.e1–9.

76. Sites CK, Cooper BC, Toth MJ, *et al.* (2007) Effect of a daily supplement of soy protein on body composition and insulin secretion in postmenopausal women. *Fertil Steril* **88**, 1609–1617.

77. Zhang Y-B, Chen W-H, Guo J-J, *et al.* (2013) Soy isoflavone supplementation could reduce body weight and improve glucose metabolism in non-Asian postmenopausal women--a meta-analysis. *Nutr Burbank Los Angel Cty Calif* **29**, 8–14.

78. Akhlaghi M, Zare M & Nouripour F (2017) Effect of Soy and Soy Isoflavones on Obesity-Related Anthropometric Measures: A Systematic Review and Meta-analysis of Randomized Controlled Clinical Trials. *Adv Nutr* **8**, 705–717.

79. Orellana-Escobedo L, Garcia-Amezquita LE, Olivas GI, et al. (2013) Capsaicinoids content and proximate composition of Mexican chili peppers (Capsicum spp.) cultivated in the State of Chihuahua. *CyTA - J. Food*, 179–184.

80. Scientific Committee on Food (2002) *Opinion of the Scientific Committee on Food on Capsaicin*. European Commision Health&Consumer protection directorate-general Brussel, Belgium.

81. Ohnuki K, Haramizu S, Oki K, *et al*. (2001) Administration of capsiate, a non-pungent capsaicin analog, promotes energy metabolism and suppresses body fat accumulation in mice. *Biosci Biotechnol Biochem* **65**, 2735–2740.

82. Kawabata F, Inoue N, Masamoto Y, *et al.* (2009) Non-pungent capsaicin analogs (capsinoids) increase metabolic rate and enhance thermogenesis via gastrointestinal TRPV1 in mice. *Biosci Biotechnol Biochem* **73**, 2690–2697.

83. Baskaran P, Krishnan V, Fettel K, *et al*. (2017) TRPV1 activation counters diet-induced obesity through sirtuin-1 activation and PRDM-16 deacetylation in brown adipose tissue. *Int. J. Obes. 2005* **41**, 739–749.

84. Baskaran P, Krishnan V, Ren J, *et al*. (2016) Capsaicin induces browning of white adipose tissue and counters obesity by activating TRPV1 channel-dependent mechanisms. *Br J Pharmacol* **173**, 2369–2389.

85. Kang J-H, Goto T, Han I-S, *et al*. (2010) Dietary capsaicin reduces obesity-induced insulin resistance and hepatic steatosis in obese mice fed a high-fat diet. *Obes Silver Spring Md* **18**, 780–787.

86. Yoshida T, Yoshioka K, Wakabayashi Y, *et al.* (1988) Effects of capsaicin and isothiocyanate on thermogenesis of interscapular brown adipose tissue in rats. *J Nutr Sci Vitaminol (Tokyo)* **34**, 587–594.

87. Masuda Y, Haramizu S, Oki K, *et al*. (2003) Upregulation of uncoupling proteins by oral administration of capsiate, a nonpungent capsaicin analog. *J Appl Physiol Bethesda Md 1985* **95**, 2408–2415.

88. Henry CJ & Emery B (1986) Effect of spiced food on metabolic rate. *Hum Nutr Clin Nutr* **40**, 165–168.

89. Yoshioka M, Lim K, Kikuzato S, *et al.* (1995) Effects of red-pepper diet on the energy metabolism in men. *J Nutr Sci Vitaminol (Tokyo)* **41**, 647–656.

90. Yoshioka M, St-Pierre S, Drapeau V, *et al.* (1999) Effects of red pepper on appetite and energy intake. *Br J Nutr* **82**, 115–123.

91. Smeets AJ & Westerterp-Plantenga MS (2009) The acute effects of a lunch containing capsaicin on energy and substrate utilisation, hormones, and satiety. *Eur J Nutr* **48**, 229–234.

92. Galgani JE & Ravussin E (2010) Effect of dihydrocapsiate on resting metabolic rate in humans123. *Am J Clin Nutr* **92**, 1089–1093.

93. Ludy M-J, Moore GE & Mattes RD (2012) The Effects of Capsaicin and Capsiate on Energy Balance: Critical Review and Meta-analyses of Studies in Humans. *Chem Senses* **37**, 103–121.

94. Janssens PLHR, Hursel R, Martens EAP, *et al.* (2013) Acute Effects of Capsaicin on Energy Expenditure and Fat Oxidation in Negative Energy Balance. *PLoS ONE* **8**. e67786

95. Snitker S, Fujishima Y, Shen H, *et al.* (2009) Effects of novel capsinoid treatment on fatness and energy metabolism in humans: possible pharmacogenetic implications. *Am. J Clin Nutr* **89**, 45–50.

96. Sun L, Camps SG, Goh HJ, *et al*. (2018) Capsinoids activate brown adipose tissue (BAT) with increased energy expenditure associated with subthreshold 18-fluorine fluorodeoxyglucose uptake in BAT-positive humans confirmed by positron emission tomography scan. *Am J Clin Nutr* **107**, 62–70.

97. Yoneshiro T, Aita S, Kawai Y, *et al.* (2012) Nonpungent capsaicin analogs (capsinoids) increase energy expenditure through the activation of brown adipose tissue in humans. *Am J Clin Nutr* **95**, 845–850.

98. Neag MA, Mocan A, Echeverría J, *et al.* (2018) Berberine: Botanical Occurrence, Traditional Uses, Extraction Methods, and Relevance in Cardiovascular, Metabolic, Hepatic, and Renal Disorders. *Front Pharmacol* **9**, 557.

99. Lee YS, Kim WS, Kim KH, *et al.* (2006) Berberine, a natural plant product, activates AMP-activated protein kinase with beneficial metabolic effects in diabetic and insulin-resistant states. *Diabetes* **55**, 2256–2264.

100. Zhang Z, Zhang H, Li B, *et al*. (2014) Berberine activates thermogenesis in white and brown adipose tissue. *Nat Commun* **5**, 5493.

101. Li Y, Wong K, Giles A, *et al.* (2014) Hepatic SIRT1 attenuates hepatic steatosis and controls energy balance in mice by inducing fibroblast growth factor 21. *Gastroenterology* **146**, 539-549.e7.

102. Wu L, Xia M, Duan Y, *et al*. (2019) Berberine promotes the recruitment and activation of brown adipose tissue in mice and humans. *Cell Death Dis* **10**, 468.

103. Yan H-M, Xia M-F, Wang Y, *et al*. (2015) Efficacy of Berberine in Patients with Non-Alcoholic Fatty Liver Disease. *PloS One* **10**, e0134172.

104. Zhang Y, Li X, Zou D, *et al*. (2008) Treatment of type 2 diabetes and dyslipidemia with the natural plant alkaloid berberine. *J Clin Endocrinol Metab* **93**, 2559–2565.

105. Yin J, Xing H & Ye J (2008) Efficacy of berberine in patients with type 2 diabetes mellitus. *Metabolism* **57**, 712–717.

106. Hu Y, Ehli EA, Kittelsrud J, *et a*l. (2012) Lipid-lowering effect of berberine in human subjects and rats. *Phytomedicine Int J Phytother Phytopharm* **19**, 861–867.
