# Supplementary material for: Feeding brown fat: dietary phytochemicals targeting non-shivering thermogenesis to control body weight
Source: Proc Nutr Soc. 2020 Apr 15;79(3):338–56. doi: 10.1017/S0029665120006928 (PMC7663322; doi:10.1017/S0029665120006928)
Supplement: Supplementary file 1 [file S0029665120006928sup.zip › S0029665120006928sup002.docx]

Table 1. Overview of rodent studies, clinical trials and *in vitro* assays addressing BAT-mediated thermogenesis by phytochemicals and their impact on energy expenditure and weight control.

| ***Pterostilbene*** | blueberries, berries, wine, wine grapes, sorghum^(1)^ | | | |
| --- | --- | --- | --- | --- |
| Reference and model | Design | Sex and N | Treatment | Outcome |
| ^(2)^  Golden Syrian Hamsters | DI | male  n=8-10 per group | 2.5 mg/kg BW/d in HFD  3 weeks | -BW plasma ↓C, ↓LDL, ↓LDL:HDL |
| ^(2)^  rat hepatic H4IIEC3 cells |  | male | 1,10,100 or 300 µM PTS  30 min | ↑PPARα reporter activity |
| ^(3)^  Zucker fa/fa rats | DI | male  n=10 per group | 15 mg/kg BW/d in HFD 6 weeks | ↓BW, ↓fat mass  plasma ↓C, ↓insulin |
| ^(4)^  Wistar rats | DI | male  n=9 per group | 15 or 30 mg/kg BW/d in HFHS  6 weeks | ↓BW gain,  ↓total fat mass, ↓vWAT, ↓sWAT  ↑hepatic CPT1α and ACOX activity |
| ^(5)^  Zucker fa/fa rats | orogastric catheter | male  n=10 per group | 0, 15 or 30 mg/kg BW/d  in chow diet,  6 weeks | ↓BW, ↓AT weight  ↑iBAT mRNA/protein: NRF1, UCP1, PPARα  ↑iBAT CPT1b activity |
| ^(6)^  OLETF rats | DI | male n=6 per group | 300 mg/kg BW/d in chow 4 weeks, | -BW, ↓abdominal WAT, ↓total WAT  ↓RER, ↑FO, ↑EE  ↓WAT FAS mRNA |
| ^(7)^  C57BL/6 mice | DI | male and female  n=8 per group/sex | 90 mg/kg BW/d in HFD 30 weeks | ↓BW, ↑glucose tolerance (after 18 weeks) ↑iWAT thermogenic genes (PPARγ, PGC1α, SIRT1, CIDEA, TBX1), ↑iWAT UCP1 protein   - effects in m+f, stronger than in f vs m |
| ^(7)^ 3T3-L1 adipocytes | acute | male | 5 µM PTS for 24 hours, d12 | ↑UCP1 protein, ↑CIDEA, FGF21 mRNA |
| ^(8)^  hypercholesterolaemic  Caucasian, AA | placebo  parallel | male and female  100 mg/d, n=20 (m5/f15)  250 mg/d, n=20 (m6/f14)  placebo, n=20  (m7/f13) | 100 or 250 mg/d   - 1. weeks | -BMI (if stratified for C medication ↓BMI)  plasma ↑total C, ↑LDL, -HDL |
| ***Resveratrol*** | peanuts and peanut products, grapes, red wine, soy, herbal remedies^(9)^ | | | |
| ^(10)^  C57Bl/6J mice | DI | male  n=8-10 per group | 400 mg/kg BW/d in HFD  15 weeks | ↓weight gain, ↓final BW, ↓WAT mass  ↑VO_2_, ↑cold-resistance (rectal T)  ↑iBAT mito content, ↑iBAT thermogenic genes (UCP1, PGC1α, PPARα)  ↑mito gene enrichment in muscle, ↑IS |
| ^(11)^  C57BL/6NIA | DI | male  n=6-9 per group | 22.4 mg/kg BW/d in HFD  middle aged mice,  for 55 weeks | -fat distribution, -BW (trend for↓), ↑IS, -BT ↑survival  plasma -TAG, ↑C, ↓GLC, ↓insulin |
| ^(12)^  grey mouse lemur (*Microcebus murinus*) | DI  Before-after | male  n=6 | 200 mg/kg BW/d  4 weeks | ↑RER, ↓weight gain vs baseline week  ↓food intake, -locomotor activity |
| ^(13)^  mice (strain unknown) | DI | male  n=8 per group | 0.4% w/w in chow diet  8 weeks | ↓WAT mass, -BW, ↑VO_2_, -locomotor activity  ↑iBAT thermogenic genes (UCP1, PRDM16, SIRT1), plasma -C, -TAG, -GLC |
| ^(14)^  CD-1 mice | DI | female  n=6 per group | 0.1% w/w in HFD  4 weeks | ↓BW gain, ↑VO_2,_ ↓RER, ↑EE (p=0.065),  ↑UCP1+ in iWAT sections, ↓iWAT adipocyte size  ↑pAMPK, UCP1, PRMD16 protein in iWAT  plasma ↓insulin, ↓TAG, -GLC |
| ^(14)^  SVF from iWAT of CD-1 mice | long-term | unknown | 10 µM RSV  during differentiation with brown adipogenic cocktail | ↑thermogenic genes (UCP1, ELOVL3, PGC1α, CIDEA, PRDM16), ↑UCP1, PRDM16 protein ↑respiration, ↑pAMPK   - effects AMPK-dependent |
| ^(15)^  CD-1 mice | DI | female | 0.1% RSV w/w in HFD  4 weeks | ↓BW gain, ↑BA number in iBAT sections  ↑iBAT UCP1. PRDM16 and pAMPK protein |
| ^(16)^  Spargue-Dawley rats | DI | male  n=8 per group | 30 mg/kg BW/d in HFHS  6 weeks | -BW, ↓fat mass  ↑BAT thermogenic genes (UCP1, PGC1α, TFAM)  ↑iBAT UCP1 protein, ↓acetylated PGC1α (muscle) |
| ^(17)^  obese, healthy (BMI>30)  Denmark | placebo  parallel | male  n=12 per group | 500 mg RSV or placebo tablet/d  4 weeks | -BW, -total fat mass, -vWAT mass,  plasma –GLC, -C, -TAG,-ALT, -HbA1c  -acetylated lysine, pAMPK in muscle |
| ^(18)^  non-obese, normoglycaemic  Caucasian | placebo  parallel | female  RSV n=15  placebo n=14 | 75 mg RSV/d  12 weeks | -BW, -fat mass, -sWAT mass, -vWAT mass  plasma -leptin,-C, -TAG, -NEFA, -GLC  -REE, -BP, -IS  -WAT microarray: -mito function, -FO genes  -muscle SIRT1 activity |
| ^(19)^  older adults with IGT  US | before-after | male n=3  female n=7 | 1, 1.5 or 2 g RSV/d  4 weeks | -BW, -fat mass, -BP  plasma –C, -TAG, -insulin, -ALT, -insulin  ↓post-meal GLC, ↓post-meal insulin |
| ^(20)^  obese, healthy men | placebo  crossover | male n=11 | 150 mg RSV/d (resVida) or placebo,  30 days per treatment | -BW, -fat mass  ↓SEE (caloric restriction), ↑diurnal RER  muscle ↑pAMPK, ↑mito activity, ↑SIRT1, PGC1α protein |
| ***Quercetin*** | apples, onions, black currants, red wine, black tea, nuts, seeds, shallots^(21)^ | | | |
| ^(22)^  Sprague–Dawley rats | DI | male  n=7 per group | 0.36% or 0.72% w/w OPE in HFD, 8 weeks | ↓BW, ↓intra-abdominal fat mass |
| ^(23)^  Wistar rats | DI | male  n=12 per group | 185, 270, 925 mg/kg BW/d in HFD  8 weeks | ↓BW gain, ↓total fat mass, ↓vWAT  plasma ↓TAG, ↓NEFA, -C, ↓GLC at high dose  ↓hepatic fat content, ↑fecal lipids  ↑PPARα, ↑SIRT1, ↓ACC, ↓FAS mRNA (WAT) |
| ^(24)^  Zucker fa/fa rats | oral gavage | male  n=7 per group | 10 mg/kg BW/d RSV or vehicle with HFD  10 weeks | ↓BW gain  plasma ↓TAG, ↓C, ↓GLC, ↓insulin, ↓HOMA-IR  ↓TNFα production, iNOs protein in vWAT |
| ^(25)^  C57BL/6 | DI | male  n=6 for OPE  n=9 for control | 0.5% w/w OPE in HFD  8 weeks | -BW gain, -eWAT mass, -rWAT mass  ↑thermogenic genes (UCP1, PRDM16, CIDEA, PGC1α) in rWAT |
| ^(25)^  3T3-L1 adipocytes | long-term | male | 100 µM quercetin at d5, 7, 9 | ↑ UCP1, SIRT1, PGC1α mRNA and protein  ↑pAMPK, pHSL, ↓lipogenic genes (FAS, ACC) |
| ^(26)^  C57BL/6 | DI | male  n=8-10 per group | 0.1% w/w quercetin in HFD  8 weeks | -BW, -fat mass,  -EE, -RER, -FO, -CHO, ↓plasma TAG  ↑sWAT thermogenic genes (UCP1, ELOVL3)  ↑UCP1+ cells in sWAT, ↑FA uptake in sWAT  -mito content sWAT, -BAT morphology/genes |
| ^(27)^  C57BL/6J | DI | male  n=8 per group | 0.8% w/w in HFD  8 weeks | -BW, -adiposity, -EE, -RER  ↓plasma inflammatory cytokines (INFγ, IL1, IL4) |
| ^(28)^  C57BL/6 | DI | male  n=6 per group | 0.05% w/w quercetin in HFD  9 weeks | -BW, ↓WAT adipocyte size  ↑UCP1+ cells, ↑UCP1, ↑PGC1α protein in iWAT  ↑iWAT thermogenic genes (UCP1, PRDM16, MEM26, NRF-1)  ↑PKA, pAMPK protein in iWAT  ↑plasma NE, ↑iBAT UCP1 |
| ^(29)^  isolated Wistar rat adipocytes | acute | male | 0, 1, 10, 100 or 250 µM quercetin, 15 min | ↑PDE activity, ↑cAMP, ↑epinephrine-stimulated lipolysis |
| ^(30)^  C57BL/6 | DI | male  n=8 per group | 0.1% w/w quercetin in HFD  12-17 weeks | ↓BW, ↓sWAT mass, ↓eWAT mass, ↓eWAT cell size, plasma ↓leptin, ↓insulin, ↓TNFα, ↓IL6  ↓mast cell infiltration eWAT  ↓eWAT TNFα, IL6  ↑eWAT SIRT1, pAMPK protein  ↑iBAT UCP1 mRNA, |
| ^(31)^  university students  healthy  Korean | placebo  crossover | female  n=12 | 100 mg/d quercetin or placebo capsule for 2 weeks each | -BW, -fat mass, -WHR, -BMI, -SBP, -DBP  plasma –TAG, -C, -LDL |
| ^(32)^  overweight/obese subjects  (BMI>23 kg/m^2^)  Korean | placebo  crossover | female  quercetin n=18  placebo n=19 | 100 mg/d quercetin or placebo capsules  12 weeks | -BW, -fat mass, -BMI  plasma –ALT,-leptin, ↑adiponectin, -TNFα, -IL4 |
| ^(33)^  overweight/obese subjects  Korean | placebo  crossover | male n=5  female n=31 | 100 mg/d quercetin or placebo capsules  12 weeks | -BW, -fat mas, -BMI, -WC, -RER, -REE  plasma –GLC, -C, -LDL, ↓TAG, -leptin  -before-after effects on REE, BW, RER, BMI within quercetin group |
| ^(34)^  Meta-analysis | 9 RCTs | male=189  female=336 | 100 to 1000 mg/d  2 to 12 weeks | -BW, -WC, -WHR, -BMI |
| ***Luteolin*** | peppers, carrots, cucumber, pomegranate, herbal spices, cabbage, broccoli, medicinal herbs (sage)^35^ | | | |
| ^(36)^  C57BL/6 | DI | male  n=8 per group | 0.01% w/w in HFD  12 weeks | ↓BW, ↓sWAT mass, ↓vWAT mass, ↓BAT mass  ↓eWAT adipocyte size, ↑IS  ↓mast cell infiltration eWAT  plasma –insulin, ↓leptin, ↑adiponectin |
| ^(37)^  C57BL/6 | DI | male  n=13 per group | 0.005% w/w in HFD  16 weeks | ↓BW, ↓sWAT mass, ↓vWAT mass  plasma ↓TAG, ↓C, ↓NEFA, ↑IS  ↑eWAT FAO genes (PGC1α, ADRB3, CPT2, PNP2, ACAD) |
| ^(38)^  C57BL/6 | DI | male  n=8 per group | 0.01% w/w in HFD  20 weeks | ↓BW, ↑IS  ↓plasma MCP1, IL6, TNFα  ↓macrophage infiltration vWAT, ↓M1/M2-ratio |
| ^(39)^  C57BL/6 | DI | male  n=12 per group | 0.01% w/w in HFD  12 weeks | ↓BW, ↓weight gain, ↓fat mass  ↑VO_2_, ↑CO2, ↑RER, ↑BAT UCP1 protein  ↑UCP1+ cells in sWAT, ↑thermogenic genes (PGC1α, UCP1, SIRT1, PPARα, ELOVL3)  ↑SIRT1, pAMPK, pACC protein in BAT, sWAT |
| ^(39)^  primary subcutaneous and brown adipocytes | acute | unknown | 24 hours of 100 nM luteolin on differentiated cells | ↑SIRT1, UCP1, PGC1α protein  ↑pAMPK, ↑pACC  ↑thermogenic genes (UCP1, PRDM16, ELVOL6, PPARα)   - effects AMPK-dependent |
| ***Catechins*** | grapes, apples, strawberries, apricots, broad beans, cocoa-products, green/black/oolong tea^(40,41)^ | | | |
| ^(42)^  Sprague-Dawley rat | DI | male  n=8 per group | 2% w/w green tea extract in HFD, 2 weeks | -BW, ↓fat mass, ↑BAT weight  ↑BAT DNA/protein content  ↑EE, propranolol prevented ↑EE |
| ^(43)^  Sprague-Dawley rat | DI | male  n=8 per group | 0.5% w/w catechins in chow  8 weeks | -BW  ↑BAT mass, ↓pWAT mass, ↓eWAT mass  ↑BAT UCP1 expression  plasma ↓TAG, GLC, leptin |
| ^(44)^  New Zealand black mice | gavage  short-term | male  n=6 per group | 3x 500 mg/kg EGCG or placebo, chow diet | -BW, -fat mass  -EE, ↓RER (p=0.053), -activity |
| ^(44)^  New Zealand black mice | DI | male  n=11 per group | 0.1% w/w EGCG in HFD  DIO 4 weeks, DI 4 weeks | ↓BW, ↓fat mass, -food intake, ↓eWAT weight plasma ↓TAG, -NEFA  -UCP1 mRNA in BAT |
| ^(45)^  iBAT depots from Sprague-Dawely rat | acute | male | 100 or 200 µM green tea extract for 40-90 min | ↑iBAT respiration (100 µM)  ↑norepinephrine (0.1 µM) stimulated respiration at 100 or 200 µM |
| ^(46)^  healthy men  Geneva, CH | placebo  crossover | male  n=10 | 3x daily capsule with 50mg caffeine and 80 mg EGCG, 50 mg caffeine or placebo | ↑diurnal EE, ↑total EE, -nocturnal EE  ↓total, diurnal and nocturnal RER  ↑FO, ↑urinary norepinephrine excretion |
| ^(47)^  young, healthy subjects  Lausanne, CH | placebo  crossover | male n=15  female n=16 | 3x daily beverage with 100 mg caffeine and 180 mg catechins or placebo, 3 days | ↑total EE, diurnal EE, nocturnal EE  -substrate oxidation  -catecholamine secretion |
| ^(48)^  healthy men  (BMI 23-27 kg/m^2^)  Laval University, CA | placebo  crossover | male  n=14 | 3x daily capsule with 200 mg caffeine plus 90, 200, 300 or 400 mg EGCG or placebo | ↑total EE, -SEE  -RER,-FO  -catecholamine secretion |
| ^(49)^  overweight/obese men  (BMI=31 kg/m^2^)  Berlin, DE | placebo  crossover | male  n=10 | 300 or 600 mg EGCG or placebo capsule for 3 days | -EE (pre- and post-meal)  ↓post-meal RQ, ↑post-meal FO (300 mg),  ↓post-meal CHO (300 mg)  plasma -NEFA,-insulin, GLC |
| ^(50)^  Meta-analysis  effect of EGCG on EE or anthropometric measures | 8 RTC | n=268 | EGCG:  300 or 600 mg/d for 2-3 days  300 to 800 mg/d for 2-12 weeks | ↑EE, ↓RER, -FO, - BMI, ↓WC, -fat percentage |
| ^(51)^  healthy men  Japanese | placebo  crossover | male n=15  low BAT activity (mean SUV=1.9) | 615 mg catechins plus 77 mg caffeine or placebo (81 mg caffeine), 2x daily as beverage for 5 weeks | -BMI, -fat mass, -WC, -EE  ↑cold-induced thermogenesis, ↑cold-induced FO |
| ^(51)^  healthy men  Japanese | acute  crossover | male n=15 | 615 mg catechins plus 77 mg caffeine or placebo (81 mg caffeine), single beverage | ↑post-drink EE,  ↑EE in high BAT (SUV>2) vs low BAT subjects, pre-assessed by 2 hours cold-exposure |
| ^(52)^  healthy university students  Japanese | placebo  parallel | female  catechin n=10  placebo n=11 | 640 mg catechins/d or placebo, beverage  12 weeks | -BMI, -fat mass, -BW  ↑BAT density in supraclavicular region  neg. correlation between EMCL and BAT density |
| ^(53)^  overweight/obese children  Japanese | placebo  parallel | catechin group  (m21, f5)  placebo group  (m13, f6) | 576 mg/d catechins or placebo (75 mg/d catechins),  as Oolong tea, 12weeks | -no changes in anthropometric or metabolites in catechin vs control  ↓WC, ↓SBP, ↓LDLC in catechin group when stratified to baseline values |
| ^(54)^  normal to overweight men  Japanese^(36)^ | placebo  parallel | male  catechin group n=17  placebo group n=19 | 690 mg/d catechins or placebo (22 mg/d),  as Oolong tea, 12weeks | ↓WC, ↓skinfold thickness, ↓total fat area  ↓visWAT and sWAT area  plasma –NEFA, -TAG, -C, -GLC, -insulin |
| ^(55)^  obese adult Thais  (BMI>25kg/m^2^) | placebo  parallel | catechin group:  (m21, f9)  placebo group:  (m21, f9) | 3x daily 250 mg catechins or placebo in capsule  12 weeks | ↓BMI, ↓BW, ↓fat mass, ↓WC, -HC  ↓RER, ↑REE |
| ^(56)^  overweight/obese adults  (BMI >25-32 kg/m^2^)  Caucasian | before-after | female n=63  male n=7 | 270 mg/d EGCG in capsule | -BW, ↓WC  -SBP, -DBP  plasma -C |
| ^(57)^  Meta-analysis  effect of green tea extracts on anthropometry | 15 RTCs | n=1243 | catechin intake combined with caffeine intake (141 up to 1207 mg/d)  8 to 24 weeks | ↓BMI, ↓BW, ↓WC, -WHR when compared to caffeine-intake only |
| ***Phytoestrogens*** | kidney beans, mung bean sprouts, Japanese arrowroot, soybean, soy products (tofu, soy milk, soy flour, soy sauce)^(58)^ | | | |
| ^(59)^  C57/B6J mice | DI | male  n=7-8 | 5% isoflavone-rich fraction of *Puerariae* flower in HFD,  7 weeks | ↓BW, ↓WAT mass, ↓BAT mass  -food intake, -fecal lipid content  ↑VO_2_, -RER, ↑UCP1+ cells in BAT sections |
| ^(60)^  CD-1 mice | DI | male n=12  female n=12 | 25% w/w soy-rich diet (150 pmm daidzein, 190 ppm genistein) vs soy-free diet  16 weeks | ↓BW, ↓intra-abdominal fat mass, ↓iWAT, ↓eWAT/ovWAT, ↓WA adipocyte size  ↓BAT mass (male), ↑brown appearance, ↓lipid droplet size  ↑cold-resistance (rectal T), ↑VO_2_, ↓RER (only male data available) |
| ^(61)^  Sprague-Dawley rats | ovx  DI | female  n=10 per group | isoflavone-rich (200 µg/g) or isoflavone-free diet  13 days | ↓BW gain, ↓abdominal fat mass  ↓serum leptin |
| ^(62)^  Long-Evans rats | DI | male | isoflavone-rich (600 ppm) or isoflavone-free (10-15 ppm) diet  up to 75 days of age | ↑food intake, ↓BW gain, ↓WAT mass, ↓BAT mass  plasma ↑T3, ↓insulin, leptin  ↑UCP1 protein in BAT |
| ^(63)^  Wistar rats | DIO with DI | male  n=16 per group | 50 mg/kg BW daidzein or vehicle, i.p.  DIO 10 weeks, 14 d treatment | ↓caloric intake, ↓BW gain  ↓hepatic liver content  plasma ↓TAG, -C, ↑GLC, -ALT  ↑UCP1 protein in BAT |
| ^(64)^  ICR mice | DIO with gavage | male | 0, 25, 50 or 100 mg/kg BW  DIO 8 weeks, 30 d treatment | ↓BW. ↓vWAT mass, ↓sWAT mass  plasma ↓C, ↓LDL, ↓NEFA, -TAG, ↑HDL |
| ^(64)^  primary adipocytes differentiated from eWAT SVF | acute | male | 0, 1, 3, 16, 64 µM daidzein  24 hours | ↑glycerol release (dose-dependent) |
| ^(65)^  adipocytes from Wistar rats | acute | male | 0.01, 0.1 or 1 mM daidzein | ↑basal lipolysis (dose-dependent)  ↑epinephrine-stimulated lipolysis (0.1 mM)  ↓lipogenesis from GLC (0.1 and 1 mM) |
| ^(66)^  C57BL/6 | DI | female  n=8 per group | 0.25% w/w genistein in HFD  8 weeks | ↓BW, -sWAT weight, -vWAT weight  ↓BAT weight (ns), ↑IS  plasma –TAG, -C, HDL-, ↓LDL, ↓NEFA  ↑iWAT browning (UCP1, CIDEA mRNA)  ↑ hypothalamic UNC3 mRNA |
| ^(67)^  C57BL/6 | DI | male  n=7-8 per group | 0.2% w/w genistein in casein diet or casein only (control)  60 days | -BW, ↑glucose tolerance  ↑thermogenic genes in sWAT (UCP1, PGC1α)  ↑UCP1 protein in sWAT,-BAT  ↑EE, ↑VO_2_, ↑cold-resistance (rectal T), -RER  plasma –TAG, ↓GLC, ↓insulin |
| ^(67)^  primary adipocytes from iWAT of mice | acute | unknown | 0, 5, 15, 30 µM genistein for 1 hour | -basal respiration  ↑maximal respiration |
| ^(68)^  immortalized brown adipocytes | long-term | unknown | 0, 0.1, 1 or 40 µM of genistein on differentiated adipocytes, 3 days treatment | ↑UCP1 promoter activity (luciferase)  ↑UCP1 activity (immunofluorescence intensity) |
| ^(69)^  C57BL/6 | oral gavage | male and female | 50 to 200 mg/kg BW genistein or vehicle for 15 d | ↓BAT mass, ↓eWAT (m), ↓abdominal WAT (f)  plasma ↓TAG, ↓C for 50 mg/kg BW |
| ^(70)^  postmenopausal women (BMI=23.6 kg/m^2^)  Chinese, equol-producer | placebo  parallel | female  n=90 per group | 40 g soy flour, 40 g low-fat milk powder with 63 mg daidzein, 40 g low fat milk powder (placebo)  daily, 6 months | -BW, -BMI, -WC, -HC, -WHR, -fat mass |
| ^(71)^  adolescent males  Tasmania | placebo  parallel | male  isoflavone n=69  placebo n=59 | 50 mg isoflavone equivalents or placebo tablets daily  6 weeks | -BW |
| ^(72)^  obese women (20-65 yrs)  (BMI 30-40 kg/m^2^)  USA | placebo  parallel | female  soy group n=22  casein group n=21 | 3x daily soy (50 mg isoflavone) or casein (3.5 mg isoflavone) shake,  16 weeks | -WC, -weight loss, -fat mass, -truncal fat  -SBP, -DBP |
| ^(73)^  impaired glycemic control  Chinese women (30-70 yrs) | placebo  parallel | female  daidzein n=55  genistein n=56  placebo n=54 | 10 g soy protein with no addition, 50 mg daidzein or 50 mg genistein  24 weeks | -BMI, -WC, -fat mass  -IS |
| ^(74)^  patients with NAFLD  Iranian (16-69 yrs) | placebo  parallel | genistein group (m30, f11)  placebo group  (m31, f10) | 250 mg daidzein or placebo capsules  8 weeks | -BW, ↓fat percentage, ↓WHR, ↓WC, -BMI  plasma ↓TAG, -C, -LDL, -HDL, ↓insulin  ↓HOMA-IR |
| ^(75)^  postmenopausal women  (BMI>30 kg/m^2^)  Caucasian or AA | placebo  parallel | soy group (n=17)  8 AA, 9 Caucasian  placebo (n=16)  8 AA, 8 Caucasian | soy protein with isoflavones (160 mg) or placebo casein, shake  3 months | -BW, -total fat, -lean mass  ↓abdominal, ↓subcutaneous abdominal fat, ↓vWAT  for AA: weight loss more than for Caucasian  for Caucasian: vWAT loss bigger than for AA  plasma ↓IL6, -CRP, -TNFα, -leptin, -HDL, -LDL,  -C, -TAG |
| ^(76)^  postmenopausal women  (mean BMI=30.5)  Caucasian | placebo  parallel | female  soy group n=9  placebo n=6 | soy protein with isoflavones (160 mg) or placebo casein,  shake  3 months | -BMI, -BW, -total fat mass, -IS  ↓subcutaneous abdominal fat, ↓intra-abdominal fat  plasma -GLC, -insulin |
| ^(77)^  Meta-analysis  Effect of soy-isoflavones on BW in non-Asian, postmenopausal women | 9 RCTs | isoflavones n=272  placebo n=256 | 40 to 160 mg/d of isoflavones  8 weeks to 1 year | ↓BW with isoflavone intake  <100 mg or <6 months more effective  more effective with BMI<30 kg/m^2^ |
| ^(78)^  Meta-analysis  effect of soy and isoflavones on anthropometric measures | 24 soy RTCs  17 isoflavones RTCs | soy:  f1265,  m45 (1 RTC)  m/f =74 (mixed)  isoflavones:  f1177, m0 | soy protein: 7.5 to 116 mg/d  4 weeks to 2 years  isoflavones: 33.3 to 300 mg/d  8 weeks to 2 years | soy:  -BW, >40 g/d ↑BW, 1-3 months ↑weight gain  -WC, -fat mass  isoflavones:  ↓BMI for postmenopausal and Caucasian women  <100 mg and 2-6 months more effective  -fat mass, -WC |
| ***Capsaicinoids*** | chili, bell peppers, jalapenos, habaneros, cayenne pepper, red pepper^(79,80)^ | | | |
| ^(81)^  Std ddY mice | intragastric tube | unknown  n=6-8 | vehicle, 10 mg/kg BW capsaicin or 10, 50 mg/kg BW capsiate, 2 weeks | ↓BW (ns), -food intake  -BAT mass, ↓eWAT for capsiate, ↓pWAT for capsaicin and 50 mg/kg capsiate |
| ^(81)^  Std ddY mice | intragastric tube, acute | unknown  n=6-8 | Vehicle, 10 mg/kg BW capsaicin or 10 mg/kg BW capsiate, 3 hours | ↑VO_2_ for capsaicin and capsiate,  ↑serum adrenaline  plasma ↑NEFA, ↓TAG |
| ^(82)^  C57BL/6 or TRPV1 -/- mice | intragastric tube, acute | male  n=5-18 | vehicle, 10 mg/kg BW capsaicin or 10 mg/kg BW capsiate, 3 hours | ↑VO_2_ (capsaicin, capsiate at 10 mg/kg BW)  ↑FO (capsaicin, capsiate at 10 mg/kg BW), ↓CHO  ↑BAT and colonic T (50 mg/kg capsinoids, 10 mg/kg capsaicin)  ↓T increase after denervation of jejunal nerves at 50 mg/kg capsinoid   - effects in wt but not TRPV-/- mice |
| ^(83)^  TRPV1 -/- or wt mice  B6.129X1 | DI | male | 0.01% w/w capsaicin in HFD  32 weeks | ↓weight gain, ↓BW,  ↑BAT UCP1, BMP8b protein  ↑activity, ↑RER, -food intake,  ↓BAT TAG content, ↑BAT glycerol release (basal or forskolin-stimulated)  ↑TRPV1 protein in BAT  ↑Ca^2+^ influx in isolated BA (2 µM CAP)  ↑pAMPK, pSIRT1 in BAT  ↓PRDM16, PPARγ acetylation (HEK293 1 µM CAP), ↑PRDM16 and PPARγ interaction in BAT lysate   - effects blunted in TRPV-/- vs wt mice |
| ^(84)^  TRPV1 -/- or wt mice  B6.129X1 | DI | male  n=40 per group | 0.01% w/w capsaicin in HFD  26 weeks | ↓weight gain,  ↑TRPV1 mRNA in iWAT, eWAT  ↑EE, ↑activity, -fecal lipid content, ↑RER, ↑VO_2_  ↑UCP1, BMP8b, PPARα/γ protein in s/eWAT,  ↑sWAT lipolysis (basal, forskolin-stimulated)  ↓PRDM16, PPARγ acetylation in sWAT  ↑pAMPk, CaMKKII activation in sWAT  ↑Ca^2+^ influx in isolated WA (2 µM CAP)   - effects blunted in TRPV-/- vs wt mice |
| ^(85)^  C57BL/6 | DIO+DI | male  n=6 per group | 0.01% w/w capsaicin in HFD  DIO 10 weeks, 10 week DI | ↓BW, ↓weight gain, ↑eWAT, ↓rWAT mass  -food intake, ↓WAT adipocyte size  ↑glucose tolerance, ↑adiponectin, ↓leptin  ↑TRPV1 expression WAT |
| ^(86)^  Sprague-Dawley rats | intra-  muscular | female  n=9-18 per group | 0.6, 0.7, 0.8 mg/kg BW capsaicin or DMSO, 80 to 120 min | ↑BAT and rectal T with 0.8 mg/kg BW  -BAT weight, -mito content  ↑BAT respiration |
| ^(87)^  Std ddY mice | intragastric tube | male  n=9-10 | 10 mg/kg BW capsiate or vehicle,  2 weeks | -BAT mass, ↓eWAT, ↓pWAT  ↑VO_2,_ ↑FO, ↑CHO, ↑UCP1 protein (BAT)  ↑UCP1 mRNA (eWAT, BAT), |
| ^(87)^  Std ddY mice | intragastric tube, acute | male  n=4 per group | 10 mg/kg BW capsiate or vehicle for 30 min | ↑UCP1 mRNA in BAT |
| ^(88)^  healthy young men  British | acute | male | breakfast with 3g chili sauce | ↑post-meal EE |
| ^(89)^  long distance runners  Japanese | acute  crossover | male  n=8 | breakfast with or without 10 g red pepper | ↑post-meal EE (30 min)  ↑RER, ↑CHO, ↓FO   - effect blocked by propranolol |
| ^(90)^  healthy young men  Caucasian | placebo  crossover | male  n=10 | appetizer with or without 6 g of red pepper | ↓energy intake at lunch and dinner  ↑sympathetic: parasympathetic nerve activity |
| ^(91)^  healthy lean subjects  Caucasian | placebo  crossover | male n=11  female n=19 | 1030 mg red pepper in lunch | -EE post-meal  -RER  -CHO, -FO, ↑peak plasma GLP-1,-ghrelin |
| ^(92)^  overweight subjects  (mean BMI=29.4 kg/m^2^)  Caucasian, AA, other | placebo  parallel | male (ethnicity)  Placebo (16,9,3) 3 mg (15, 9, 1)  9 mg (10, 4, 2) | 0, 3, 9 mg dihydrocapsiate in gel capsule  4 weeks | -RMR (p=0.054 for 3 mg vs placebo)  ↑RMR dihydrocapsiate vs placebo |
| ^(93)^  Meta-analysis  effect of capsaicin or capsiate on EE, RER | capsiate on EE 13 RCTs  capsiate on RER 9 RCTs  capsaicin on EE 13 RCTs  capsaicin on RER 10 RCTs  female/male unknown | | capsaicin doses: <7, 20-35 mg, 135-150 mg/d  dihydrocapsiate doses: <1.5, 2-4 or 6-9 mg/d | ↑EE at 2-9 mg/d for capsiate  ↑EE at 135-150 mg/d for capsaicin  ↑FO at 6-9 mg/d capsiate  ↑FO at 20-150 mg/d for capsaicin  ↑SNS activity (1 RCT) |
| ^(94)^  healthy adult participants (BMI 20-30)  Caucasian | acute  controlled | N=15 per group  (m8, f7) | 0 or 7.68 mg/d capsaicin with 100% or 75% of daily energy requirements  26 hours | -TEE 100% CAP vs 100% control (c)  -DIT 75% CAP vs 100% c, ↓75 % c vs 100%  -REE 75% CAP vs 100% c, ↓75 % c vs 100%  ↑FAO 75% CAP vs 100% c, -75 % c vs 100%  ↓CHO 75% CAP vs 100% c |
| ^(95)^  healthy adults  BMI 25-30 kg/m^2^  44% Hispanic, 41% white, non-Hispanic, 13% black, 2% others | placebo  parallel | capsinoids  (m21, f22)  Placebo  (m17, f20) | 6 mg capsinoids or placebo capsule with MCTG, rapeseed oil  12 weeks | -BW, -fat mass, -abdominal fat  -EE (only m): 54 kcal/d higher with CAP (p=019)  -FO (only m): 21 mg/min higher with CAP (p=0.06) |
| ^(96)^  lean, healthy subjects  Singapore | crossover  cold vs capsinoids | Male n=8,  female n=12  BAT+ m6/f6  BAT- m2/f6 | 12 mg capsinoids in capsule with rapeseed oil and MCTG | ↑FDG-uptake in BAT  ↑EE, higher in BAT+ subjects  ↑FO, ↓RER  plasma –GLC, -TAG, ↑C, ↑NEFA |
| ^(97)^  young healthy men  Japanese | placebo  crossover  acute | male n=18  BAT+ n=10  BAT- n=8 | 9 mg capsinoids or placebo capsule with rapeseed oil, MCTG  2 hours | ↑EE in BAT+ with capsinoids vs placebo  -RER, -skin T |
| ***Berberine*** | barberry, supplements from bark, root, stems or leaves from plants of the *Berberis* genus (e.g. goldenseal, goldthread, Oregon grape, tree turmeric)^(98)^ | | | |
| ^(99)^  db/db mice | i.p. | male  BBR n=17  vehicle n=16 | 5 mg/kg BW on chow diet  26 days | ↓BW, ↓eWAT mass, ↓sWAT adipocyte size ↓intra-abdominal fat, ↑IS,  ↑BAT mRNA, PPARα, PGC1α ↓FAS  ↓WAT mRNA FAS, PPARγ, SREBP1c, aP2 |
| ^(99)^  3T3-L1 adipocytes | acute | male | 5 µg/mL BBR for 60 min | ↑pAMPK, ↑pACC |
| ^(100)^  db/db mice | long-term  i.p | male  n=5 per group | 5 mg/kg BW on chow diet  26 days at 22°C or 30°C | ↓BW, ↓fat mass, ↓plasma NEFA ↑rectal T, ↑VCO_2_, ↑VO_2_, ↑EE  ↑cold-resistance (core T)  ↑BAT activity (PET/CT)  ↑BAT mito content, ↑oxphos, ↓BAT mass  ↑BAT UCP1, PGC1α, CPT1, pAMPK protein  ↑iWAT thermogenic genes (UCP1, NRF1) ↑UCP1+ cells, ↑mito content, ↑UCP1 protein   - effects blunted at 30°C |
| ^(101)^  C57BL/6 | DI | male | 5 mg/kg BW, i.p.  on chow diet, 4 weeks | ↑hepatic FGF21 expression, ↑plasma FGF21  ↑iBAT mRNA (UCP1, DIO2, PRMD16) |
| ^(102)^  C57BL/6 | DIO | male  n=7-8 | 1.5 mg/kg BW/d on HFD  DIO 8 weeks, DI 6 weeks | ↓BW gain, ↓pWAT, iWAT mass  ↑rectal T, ↑EE, ↑VO_2,_ ↑BAT activity, volume (PET/CT), ↑UCP1+ cells in BAT  ↓BAT PRDM16 promoter methylation   - effects blunted in adiponectinCre AMPKα1/2 mice |
| ^(102)^  BAT SVF cells | long-term | male | 250 nM BBR  during differentiation until d8 | ↑basal and uncoupled respiration  ↑BA adipogenesis (↑UCP1+ cells)  ↑fatty acid oxidative BAT-specific genes  ↑UCP1, PGC1α, PRDM16 protein |
| ^(102)^  patients with NAFLD  mean BMI=29 kg/m2  China | before-after | not defined | 1.5 g BBR/d  1 month | ↓BW, ↓WC, ↓BMI, -total fat mass,  ↓vWAT mass, ↓sWAT mass  ↓HOMA-IR  ↑BAT volume, BAT activity |
| ^(103)^  subjects with NAFLD  China | controlled  parallel | LSI (m32/f30) LSI+P (m28/f32) LSI+BBR (m38/f24) | LSI+1.5g BBR/d, LSI+15 mg/d pioglitazone, LSI only  16 weeks | ↓hepatic fat content vs LSI,  ↓BW, ↓BMI vs LSI and vs LSI+pioglitazone  -HbA1c, ↓HOMA-IR vs LSI, |
| ^(104)^  newly diagnosed diabetics  no pharmacotherapy  China | placebo  parallel | placebo (m38, f28)  BBR (m31, f21) | 1.5 g BBR/d or placebo  12 weeks | -BW, ↓BMI, -WHR  plasma –FBG, ↓PBG, ↓HbA1c, -HOMA-IR, ↓C,  - insulin, -TAG |
| ^(105)^  type 2 diabetics with poor glycemic control  China | before-after | n=48  sex not stated | 1.5 g BBR/d plus prescribed diabetes medication  12 weeks | ↓WC, ↓WHR, -BMI  plasma ↓FBG, ↓PBG, -TAG, ↓C, ↓insulin  ↓HOMA-IR |
| ^(106)^  obese adults  Caucasian | before-after | male n=2  female n=5 | 1.5 g BBR/d  12 weeks | -BMI, -WHR, - fat percentage  plasma –GLC,-TAG, ↓C, ↓ALT, ↓AST  -plasma inflammatory markers |

AA, African American, BAT, brown adipose tissue; BBR, berberine; BW, body weight; BMI, body mass index, C, cholesterol; CHO, carbohydrate oxidation; d, day; DI, dietary intervention; DIO, diet-induced obesity; DIT, diet-induced thermogenesis; EE, energy expenditure; EMCL, extramyocellular lipid; f, female; FBG, fasting blood glucose; FO, fat oxidation; HC, hip circumference; HFHS, high fat high sucrose; IS, insulin sensitivity, IGT, impaired glucose tolerance; i.p., intraperitoneal; LSI, life style intervention; m, male; MCTG, medium chain triglyceride; mito, mitochondria(l); NAFLD, non-alcoholic fatty liver disease, NEFA, non-esterified fatty acids; ovx, ovariectomized, PBG, postprandial blood glucose; RCT, randomized controlled trial; REE, resting energy expenditure; RER, resting energy expenditure; SEE, sleeping energy expenditure; SUV, standardized uptake value; T, temperature; TAG, triacylglycerol, TEE, total energy expenditure; WC, waist circumference; WHR, waist-hip-ratio; WAT, white adipose tissue, wt, wild-type; - unchanged, ↓decrease, ↑increase compared to placebo or baseline

References

1. Reinisalo M, Kårlund A, Koskela A, *et al.* (2015) Polyphenol Stilbenes: Molecular Mechanisms of Defence against Oxidative Stress and Aging-Related Diseases. *Oxid Med Cell Longev* **2015**, 340520.
2. Rimando AM, Nagmani R, Feller DR, *et al.* (2005) Pterostilbene, a new agonist for the peroxisome proliferator-activated receptor alpha-isoform, lowers plasma lipoproteins and cholesterol in hypercholesterolemic hamsters. *J Agric Food Chem* **53**, 3403–3407.
3. Etxeberria U, Hijona E, Aguirre L, *et al*. (2017) Pterostilbene-induced changes in gut microbiota composition in relation to obesity. *Mol Nutr Food Res* **61**, 1500906

4. Gómez-Zorita S, Fernández-Quintela A, Lasa A, *et al.* (2014) Pterostilbene, a dimethyl ether derivative of resveratrol, reduces fat accumulation in rats fed an obesogenic diet. *J Agric Food Chem* **62**, 8371–8378.

5. Aguirre L, Milton-Laskibar I, Hijona E, *et al.* (2016) Effects of pterostilbene in brown adipose tissue from obese rats. *J Physiol Biochem* **73**, 457–464.

6. Nagao K, Jinnouchi T, Kai S, *et al.* (2017) Pterostilbene, a dimethylated analog of resveratrol, promotes energy metabolism in obese rats. *J Nutr Biochem* **43**, 151–155.

7. La Spina M, Galletta E, Azzolini M, *et al.* (2019) Browning Effects of a Chronic Pterostilbene Supplementation in Mice Fed a High-Fat Diet. *Int J Mol Sci* **20**, 5377.

8. Riche DM, Riche KD, Blackshear CT, *et al*. (2014) Pterostilbene on metabolic parameters: a randomized, double-blind, and placebo-controlled trial. *Evid.-Based Complement Alter Med ECAM* ***2014***, 459165.

9. Burns J, Yokota T, Ashihara H, *et al*. (2002) Plant foods and herbal sources of resveratrol. *J Agric Food Chem* **50**, 3337–3340.

10. Lagouge M, Argmann C, Gerhart-Hines Z, *et al.* (2006) Resveratrol improves mitochondrial function and protects against metabolic disease by activating SIRT1 and PGC-1alpha. *Cell* **127**, 1109–1122.

11. Baur JA, Pearson KJ, Price NL, *et al*. (2006) Resveratrol improves health and survival of mice on a high-calorie diet. *Nature* **444**, 337–342.

12. Dal-Pan A, Blanc S & Aujard F (2010) Resveratrol suppresses body mass gain in a seasonal non-human primate model of obesity. *BMC Physiol* **10**, 11.

13. Andrade JMO, Frade ACM, Guimarães JB, *et al.* (2014) Resveratrol increases brown adipose tissue thermogenesis markers by increasing SIRT1 and energy expenditure and decreasing fat accumulation in adipose tissue of mice fed a standard diet. *Eur J Nutr* **53**, 1503–1510.

14. Wang S, Liang X, Yang Q, *et al*. (2015) Resveratrol induces brown-like adipocyte formation in white fat through activation of AMP-activated protein kinase (AMPK) α1. *Int J Obes 2005* **39**, 967–976.

15. Wang S, Liang X, Yang Q, *et al.* (2017) Resveratrol enhances brown adipocyte formation and function by activating AMP-activated protein kinase (AMPK) α1 in mice fed high-fat diet. *Mol Nut Food Res* **61**, 1600746.

16. Alberdi G, Rodríguez VM, Miranda J, *et al.* (2013) Thermogenesis is involved in the body-fat lowering effects of resveratrol in rats. *Food Chem* **141**, 1530–1535.

17. Poulsen MM, Vestergaard PF, Clasen BF, *et al*. (2013) High-Dose Resveratrol Supplementation in Obese Men. *Diabetes* **62**, 1186–1195.

18. Yoshino J, Conte C, Fontana L, *et al*. (2012) Resveratrol supplementation does not improve metabolic function in nonobese women with normal glucose tolerance. *Cell Metab* **16**, 658–664.

19. Crandall JP, Oram V, Trandafirescu G, *et al*. (2012) Pilot study of resveratrol in older adults with impaired glucose tolerance. *J Gerontol A Biol Sci Med Sci* **67**, 1307–1312.

20. Timmers S, Konings E, Bilet L, *et al.* (2011) Calorie restriction-like effects of 30 days of Resveratrol (resVida^TM^) supplementation on energy metabolism and metabolic profile in obese humans. *Cell Metab* **14,** 612-622.

21. Li Y, Yao J, Han C, *et al.* (2016) Quercetin, Inflammation and Immunity. *Nutrients* **8**, 187

22. Moon J, Do H-J, Kim OY, *et al*. (2013) Antiobesity effects of quercetin-rich onion peel extract on the differentiation of 3T3-L1 preadipocytes and the adipogenesis in high fat-fed rats. *Food Chem Toxicol Int J Publ Br Ind Biol Res Asso* **58**, 347–354.

23. Ting Y, Chang W-T, Shiau D-K, *et al*. (2018) Antiobesity Efficacy of Quercetin-Rich Supplement on Diet-Induced Obese Rats: Effects on Body Composition, Serum Lipid Profile, and Gene Expression. *J. Agric Food Chem* **66**, 70–80.

24. Rivera L, Morón R, Sánchez M, *et al.* (2008) Quercetin ameliorates metabolic syndrome and improves the inflammatory status in obese Zucker rats. *Obes. Silver Spring Md* **16**, 2081–2087.

25. Lee SG, Parks JS & Kang HW (2017) Quercetin, a functional compound of onion peel, remodels white adipocytes to brown-like adipocytes. *J Nutr Biochem* **42**, 62–71.

26. Kuipers EN, van Dam AD, Held NM, *et al.* (2018) Quercetin Lowers Plasma Triglycerides Accompanied by White Adipose Tissue Browning in Diet-Induced Obese Mice. *Int J Mol Sci* **19**.

27. Stewart LK, Soileau JL, Ribnicky D, *et al.* (2008) Quercetin transiently increases energy expenditure but persistently decreases circulating markers of inflammation in C57BL/6J mice fed a high-fat diet. *Metabolism* **57**, S39–S46.

28. Choi H, Kim C-S & Yu R (2018) Quercetin Upregulates Uncoupling Protein 1 in White/Brown Adipose Tissues through Sympathetic Stimulation. *J Obes Metab Syndr* **27**, 102–109.

29. Kuppusamy UR & Das NP (1994) Potentiation of beta-adrenoceptor agonist-mediated lipolysis by quercetin and fisetin in isolated rat adipocytes. *Biochem Pharmacol* **47**, 521–529.

30. Dong J, Zhang X, Zhang L, *et al.* (2014) Quercetin reduces obesity-associated ATM infiltration and inflammation in mice: a mechanism including AMPKα1/SIRT1. *J Lipid Res* **55**, 363–374.

31. Kim J, Cha Y-J, Lee K-H, *et al*. (2013) Effect of onion peel extract supplementation on the lipid profile and antioxidative status of healthy young women: a randomized, placebo-controlled, double-blind, crossover trial. *Nutr Res Pract* **7**, 373–379.

32. Kim K-A & Yim J-E (2016) The Effect of Onion Peel Extract on Inflammatory Mediators in Korean Overweight and Obese Women. *Clin Nutr Res* **5**, 261–269.

33. Lee J-S, Cha Y-J, Lee K-H, *et al.* (2016) Onion peel extract reduces the percentage of body fat in overweight and obese subjects: a 12-week, randomized, double-blind, placebo-controlled study. *Nut Res Pract* **10**, 175–181.

34. Huang H, Liao D, Dong Y, *et al*. (2019) Clinical effectiveness of quercetin supplementation in the management of weight loss: a pooled analysis of randomized controlled trials. *Diabetes Metab Syndr Obes Targets Ther* **12**, 553–563

35. [López-Lázaro M](https://www.ncbi.nlm.nih.gov/pubmed/?term=L%C3%B3pez-L%C3%A1zaro%20M%5BAuthor%5D&cauthor=true&cauthor_uid=19149659) (2009) Distribution and biological activities of the flavonoid luteolin. *Mini Rev Med Chem* **9***,* 31-59

36. Xu N, Zhang L, Dong J, *et al.* (2014) Low-dose diet supplement of a natural flavonoid, luteolin, ameliorates diet-induced obesity and insulin resistance in mice. *Mol Nutr Food Res* **58**, 1258–1268.

37. Kwon E-Y, Jung UJ, Park T, *et al*. (2015) Luteolin attenuates hepatic steatosis and insulin resistance through the interplay between the liver and adipose tissue in mice with diet-induced obesity. *Diabetes* **64**, 1658–1669.

38. Zhang L, Han Y-J, Zhang X, *et al*. (2016) Luteolin reduces obesity-associated insulin resistance in mice by activating AMPKα1 signalling in adipose tissue macrophages. *Diabetologia* **59**, 2219–2228.

39. Zhang X, Zhang Q-X, Wang X, *et* al (2016) Dietary luteolin activates browning and thermogenesis in mice through an AMPK/PGC1α pathway-mediated mechanism. *Int J Obes 2005* **40**, 1841–1849.

40. Henning SM, Fajardo-Lira C, Lee HW, et al. (2003) Catechin Content of 18 Teas and a Green Tea Extract Supplement Correlates With the Antioxidant Capacity. *Nutr Cancer* **45**, 226–235.

41. Arts ICW, van de Putte B & Hollman PCH (2000) Catechin Contents of Foods Commonly Consumed in The Netherlands. 2. Tea, Wine, Fruit Juices, and Chocolate Milk. *J. Agric Food Chem.* **48**, 1752–1757.

42. Choo JJ (2003) Green tea reduces body fat accretion caused by high-fat diet in rats through beta-adrenoceptor activation of thermogenesis in brown adipose tissue. *J. Nutr Biochem* **14**, 671–676.

43. Nomura S, Ichinose T, Jinde M, *et al*. (2008) Tea catechins enhance the mRNA expression of uncoupling protein 1 in rat brown adipose tissue. *J Nut. Biochem* **19**, 840–847.

44. Klaus S, Pültz S, Thöne-Reineke C, *et al*. (2005) Epigallocatechin gallate attenuates diet-induced obesity in mice by decreasing energy absorption and increasing fat oxidation. *Int J Obes 2005* **29**, 615–623.

45. Dulloo AG, Seydoux J, Girardier L, *et al.* (2000) Green tea and thermogenesis: interactions between catechin-polyphenols, caffeine and sympathetic activity. *Int J Obes Relat Metab Disord J Int Assoc Study Obes* **24**, 252–258.

46. Dulloo AG, Duret C, Rohrer D, *et al*. (1999) Efficacy of a green tea extract rich in catechin polyphenols and caffeine in increasing 24-h energy expenditure and fat oxidation in humans. *Am. J. Clin. Nutr* **70**, 1040–1045.

47. Rudelle S, Ferruzzi MG, Cristiani I, *et al*. (2007) Effect of a thermogenic beverage on 24-hour energy metabolism in humans. *Obes Silver Spring Md* **15**, 349–355.

48. Bérubé-Parent S, Pelletier C, Doré J, *et al.* (2005) Effects of encapsulated green tea and Guarana extracts containing a mixture of epigallocatechin-3-gallate and caffeine on 24 h energy expenditure and fat oxidation in men. *Br J Nutr* **94**, 432–436.

49. Thielecke F, Rahn G, Böhnke J, *et al.* (2010) Epigallocatechin-3-gallate and postprandial fat oxidation in overweight/obese male volunteers: a pilot study. *Eur J Clin Nutr* **64**, 704–713.

50. Kapoor MP, Sugita M, Fukuzawa Y, *et al*. (2017) Physiological effects of epigallocatechin-3-gallate (EGCG) on energy expenditure for prospective fat oxidation in humans: A systematic review and meta-analysis*. Nutr Biochem* **43**, 1–10.

51. Yoneshiro T, Matsushita M, Hibi M, *et al.* (2017) Tea catechin and caffeine activate brown adipose tissue and increase cold-induced thermogenic capacity in humans. *Am. J Clin Nutr* **105**, 873–881.

52. Nirengi S, Amagasa S, Homma T, *et al*. (2016) Daily ingestion of catechin-rich beverage increases brown adipose tissue density and decreases extramyocellular lipids in healthy young women. *SpringerPlus* **5**, 1363.

53. Matsuyama T, Tanaka Y, Kamimaki I, *et al*. (2008) Catechin safely improved higher levels of fatness, blood pressure, and cholesterol in children. *Obes Silver Spring Md* **16**, 1338–1348.

54. Nagao T, Komine Y, Soga S, *et al*. (2005) Ingestion of a tea rich in catechins leads to a reduction in body fat and malondialdehyde-modified LDL in men. *Am J Clin Nut.* **81**, 122–129.

55. Auvichayapat P, Prapochanung M, Tunkamnerdthai O, *et al*. (2008) Effectiveness of green tea on weight reduction in obese Thais: A randomized, controlled trial. *Physiol Behav* **93**, 486–491.

56. Chantre P & Lairon D (2002) Recent findings of green tea extract AR25 (Exolise) and its activity for the treatment of obesity. *Phytomedicine* **9**, 3–8.

57. Phung OJ, Baker WL, Matthews LJ, *et al.* (2010) Effect of green tea catechins with or without caffeine on anthropometric measures: a systematic review and meta-analysis. *Am J Clin Nutr* **91**, 73–81.

58. Zaheer K & Humayoun Akhtar M (2017) An updated review of dietary isoflavones: Nutrition, processing, bioavailability and impacts on human health. *Crit Rev Food Sci Nutr* **57**, 1280–1293.

59. Kamiya T, Nagamine R, Sameshima-Kamiya M, *et al*. (2012) The isoflavone-rich fraction of the crude extract of the Puerariae flower increases oxygen consumption and BAT UCP1 expression in high-fat diet-fed mice. *Glob J Health Sc.* **4**, 147–155.

60. Cederroth CR, Vinciguerra M, Kühne F, *et al*. (2007) A Phytoestrogen-Rich Diet Increases Energy Expenditure and Decreases Adiposity in Mice. *Environ Health Perspect* **115**, 1467–1473.

61. Russell AL, Grimes JM, Cruthirds DF, *et al*. (2017) Dietary Isoflavone-Dependent and Estradiol Replacement Effects on Body Weight in the Ovariectomized (OVX) Rat. *Horm Metab Res Horm Stoffwechselforschung Horm Metab* **49**, 457–465.

62. Lephart ED, Porter JP, Lund TD, *et al*. (2004) Dietary isoflavones alter regulatory behaviors, metabolic hormones and neuroendocrine function in Long-Evans male rats. *Nutr. Metab.* **1**, 16.

63. Crespillo A, Alonso M, Vida M, *et al*. (2011) Reduction of body weight, liver steatosis and expression of stearoyl-CoA desaturase 1 by the isoflavone daidzein in diet-induced obesity. *Br J Pharmacol* **164**, 1899–1915

64. Guo Y, Wu G, Su X, *et al*. (2009) Antiobesity action of a daidzein derivative on male obese mice induced by a high-fat diet. *Nutr Res N. Y. N* **29**, 656–663.

65. Szkudelska K, Szkudelski T & Nogowski L (2002) Daidzein, coumestrol and zearalenone affect lipogenesis and lipolysis in rat adipocytes. *Phytomedicine Int J Phytother Phytopharm* **9**, 338–345.

66. Zhou L, Xiao X, Zhang Q, *et al*. (2019) A Possible Mechanism: Genistein Improves Metabolism and Induces White Fat Browning Through Modulating Hypothalamic Expression of Ucn3, Depp, and Stc1. *Front Endocrino.* **10**, 478.

67. Palacios-González B, Vargas-Castillo A, Velázquez-Villegas LA, *et al*. (2019) Genistein increases the thermogenic program of subcutaneous WAT and increases energy expenditure in mice. *J Nutr Biochem* **68**, 59–68.

68. Buhlmann E, Horváth C, Houriet J, *et al*. (2019) Puerariae lobatae root extracts and the regulation of brown fat activity. *Phytomedicine Int J Phytother Phytopharm* **64**, 153075.

69. Penza M, Montani C, Romani A, *et al.* (2006) Genistein affects adipose tissue deposition in a dose-dependent and gender-specific manner. *Endocrinology* **147**, 5740–5751.

70. Liu Z-M, Ho SC, Chen Y-M, *et al.* (2013) A six-month randomized controlled trial of whole soy and isoflavones daidzein on body composition in equol-producing postmenopausal women with prehypertension. *J Obes* **2013**, 359763.

71. Jones G, Dwyer T, Hynes K, *et al*. (2003) A randomized controlled trial of phytoestrogen supplementation, growth and bone turnover in adolescent males. *Eur J Clin Nut.* **57**, 324–327.

72. Anderson JW, Fuller J, Patterson K, *et al.* (2007) Soy compared to casein meal replacement shakes with energy-restricted diets for obese women: randomized controlled trial. *Metabolism* **56**, 280–288.

73. Ye Y-B, Chen A-L, Lu W, *et al*. (2015) Daidzein and genistein fail to improve glycemic control and insulin sensitivity in Chinese women with impaired glucose regulation: A double-blind, randomized, placebo-controlled trial. *Mol Nut Foodes* **59**, 240–249.

74. Amanat S, Eftekhari MH, Fararouei M, *et al.* (2018) Genistein supplementation improves insulin resistance and inflammatory state in non-alcoholic fatty liver patients: A randomized, controlled trial. *Clin Nutr Edinb Scotl* **37**, 1210–1215.

75. Christie DR, Grant J, Darnell BE, *et al*. (2010) Metabolic effects of soy supplementation in postmenopausal Caucasian and African American women: a randomized, placebo-controlled trial. *Am J Obstet Gynecol* **203**, 153.e1–9.

76. Sites CK, Cooper BC, Toth MJ, *et al.* (2007) Effect of a daily supplement of soy protein on body composition and insulin secretion in postmenopausal women. *Fertil Steril* **88**, 1609–1617.

77. Zhang Y-B, Chen W-H, Guo J-J, *et al*. (2013) Soy isoflavone supplementation could reduce body weight and improve glucose metabolism in non-Asian postmenopausal women--a meta-analysis. *Nutr Burbank Los Angel Cty Calif* **29**, 8–14.

78. Akhlaghi M, Zare M & Nouripour F (2017) Effect of Soy and Soy Isoflavones on Obesity-Related Anthropometric Measures: A Systematic Review and Meta-analysis of Randomized Controlled Clinical Trials. *Adv Nutr* **8**, 705–717.

79. Orellana-Escobedo L, Garcia-Amezquita LE, Olivas GI, et al. (2013) Capsaicinoids content and proximate composition of Mexican chili peppers (Capsicum spp.) cultivated in the State of Chihuahua. *CyTA - J. Food*, 179–184.

80. Scientific Committee on Food (2002) *Opinion of the Scientific Committee on Food on Capsaicin*. European Commision Health&Consumer protection directorate-general Brussel, Belgium.

81. Ohnuki K, Haramizu S, Oki K, *et al*. (2001) Administration of capsiate, a non-pungent capsaicin analog, promotes energy metabolism and suppresses body fat accumulation in mice. *Biosci Biotechnol Biochem* **65**, 2735–2740.

82. Kawabata F, Inoue N, Masamoto Y, *et al.* (2009) Non-pungent capsaicin analogs (capsinoids) increase metabolic rate and enhance thermogenesis via gastrointestinal TRPV1 in mice. *Biosci Biotechnol Biochem* **73**, 2690–2697.

83. Baskaran P, Krishnan V, Fettel K, *et al*. (2017) TRPV1 activation counters diet-induced obesity through sirtuin-1 activation and PRDM-16 deacetylation in brown adipose tissue. *Int. J. Obes. 2005* **41**, 739–749.

84. Baskaran P, Krishnan V, Ren J, *et al*. (2016) Capsaicin induces browning of white adipose tissue and counters obesity by activating TRPV1 channel-dependent mechanisms. *Br J Pharmacol* **173**, 2369–2389.

85. Kang J-H, Goto T, Han I-S, *et al*. (2010) Dietary capsaicin reduces obesity-induced insulin resistance and hepatic steatosis in obese mice fed a high-fat diet. *Obes Silver Spring Md* **18**, 780–787.

86. Yoshida T, Yoshioka K, Wakabayashi Y, *et al.* (1988) Effects of capsaicin and isothiocyanate on thermogenesis of interscapular brown adipose tissue in rats. *J Nutr Sci Vitaminol (Tokyo)* **34**, 587–594.

87. Masuda Y, Haramizu S, Oki K, *et al*. (2003) Upregulation of uncoupling proteins by oral administration of capsiate, a nonpungent capsaicin analog. *J Appl Physiol Bethesda Md 1985* **95**, 2408–2415.

88. Henry CJ & Emery B (1986) Effect of spiced food on metabolic rate. *Hum Nutr Clin Nutr* **40**, 165–168.

89. Yoshioka M, Lim K, Kikuzato S*, et al.* (1995) Effects of red-pepper diet on the energy metabolism in men. *J Nutr Sci Vitaminol (Tokyo)* **41**, 647–656.

90. Yoshioka M, St-Pierre S, Drapeau V, *et al.* (1999) Effects of red pepper on appetite and energy intake. *Br J Nutr* **82**, 115–123.

91. Smeets AJ & Westerterp-Plantenga MS (2009) The acute effects of a lunch containing capsaicin on energy and substrate utilisation, hormones, and satiety. *Eur J Nutr* **48**, 229–234.

92. Galgani JE & Ravussin E (2010) Effect of dihydrocapsiate on resting metabolic rate in humans123. *Am J Clin Nutr* **92**, 1089–1093.

93. Ludy M-J, Moore GE & Mattes RD (2012) The Effects of Capsaicin and Capsiate on Energy Balance: Critical Review and Meta-analyses of Studies in Humans. *Chem Senses* **37**, 103–121.

94. Janssens PLHR, Hursel R, Martens EAP, *et al.* (2013) Acute Effects of Capsaicin on Energy Expenditure and Fat Oxidation in Negative Energy Balance. *PLoS ONE* **8**. e67786

95. Snitker S, Fujishima Y, Shen H, *et al.* (2009) Effects of novel capsinoid treatment on fatness and energy metabolism in humans: possible pharmacogenetic implications. *Am. J Clin Nutr* **89**, 45–50.

96. Sun L, Camps SG, Goh HJ*, et al*. (2018) Capsinoids activate brown adipose tissue (BAT) with increased energy expenditure associated with subthreshold 18-fluorine fluorodeoxyglucose uptake in BAT-positive humans confirmed by positron emission tomography scan. *Am J Clin Nutr* **107**, 62–70.

97. Yoneshiro T, Aita S, Kawai Y, *et al.* (2012) Nonpungent capsaicin analogs (capsinoids) increase energy expenditure through the activation of brown adipose tissue in humans. *Am J Clin Nutr* **95**, 845–850.

98. Neag MA, Mocan A, Echeverría J, *et al.* (2018) Berberine: Botanical Occurrence, Traditional Uses, Extraction Methods, and Relevance in Cardiovascular, Metabolic, Hepatic, and Renal Disorders. *Front Pharmacol* **9**, 557.

99. Lee YS, Kim WS, Kim KH, *et al.* (2006) Berberine, a natural plant product, activates AMP-activated protein kinase with beneficial metabolic effects in diabetic and insulin-resistant states. *Diabetes* **55**, 2256–2264.

100. Zhang Z, Zhang H, Li B, *et al*. (2014) Berberine activates thermogenesis in white and brown adipose tissue. *Nat Commun* **5**, 5493.

101. Li Y, Wong K, Giles A, *et al.* (2014) Hepatic SIRT1 attenuates hepatic steatosis and controls energy balance in mice by inducing fibroblast growth factor 21. *Gastroenterology* **146**, 539-549.e7.

102. Wu L, Xia M, Duan Y, *et al*. (2019) Berberine promotes the recruitment and activation of brown adipose tissue in mice and humans. *Cell Death Dis* **10**, 468.

103. Yan H-M, Xia M-F, Wang Y, *et al*. (2015) Efficacy of Berberine in Patients with Non-Alcoholic Fatty Liver Disease. *PloS One* **10**, e0134172.

104. Zhang Y, Li X, Zou D, *et al*. (2008) Treatment of type 2 diabetes and dyslipidemia with the natural plant alkaloid berberine. *J Clin Endocrinol Metab* **93**, 2559–2565.

105. Yin J, Xing H & Ye J (2008) Efficacy of berberine in patients with type 2 diabetes mellitus. *Metabolism* **57**, 712–717.

106. Hu Y, Ehli EA, Kittelsrud J, *et a*l. (2012) Lipid-lowering effect of berberine in human subjects and rats. *Phytomedicine Int J Phytother Phytopharm* **19**, 861–867.
